# Solvent-free preparation and thermocompression self-assembly: an exploration of performance improvement strategies for perovskite solar cells

**DOI:** 10.1039/d4ra02191f

**Published:** 2024-05-28

**Authors:** Fang Luo, Doha Lim, Hae-Jun Seok, Han-Ki Kim

**Affiliations:** a School of Advanced Materials Science and Engineering, Sungkyunkwan University 2066, Seobu-ro Jangan-gu Suwon-si Gyeonggi-do 16419 the Republic of Korea

## Abstract

Perovskite solar cells (PSCs) exhibit sufficient technological efficiency and economic competitiveness. However, their poor stability and scalability are crucial factors limiting their rapid development. Therefore, achieving both high efficiency and good stability is an urgent challenge. In addition, the preparation methods for PSCs are currently limited to laboratory-scale methods, so their commercialization requires further research. Effective packaging technology is essential to protect the PSCs from degradation by external environmental factors and ensure their long-term stability. The industrialization of PSCs is also inseparable from the preparation technology of perovskite thin films. This review discusses the solvent-free preparation of PSCs, shedding light on the factors that affect PSC performance and strategies for performance enhancement. Furthermore, this review analyzes the existing simulation techniques that have contributed to a better understanding of the interfacial evolution of PSCs during the packaging process. Finally, the current challenges and possible solutions are highlighted, providing insights to facilitate the development of highly efficient and stable PSC modules to promote their widespread application.

## Abstract 2

In this review, methods to improve the performance of PSCs and improvement principle are analyzed. The current challenges and possible solutions are proposed. It provides a new way to realize the balance between high efficiency and high stability.

## Graphical abstract

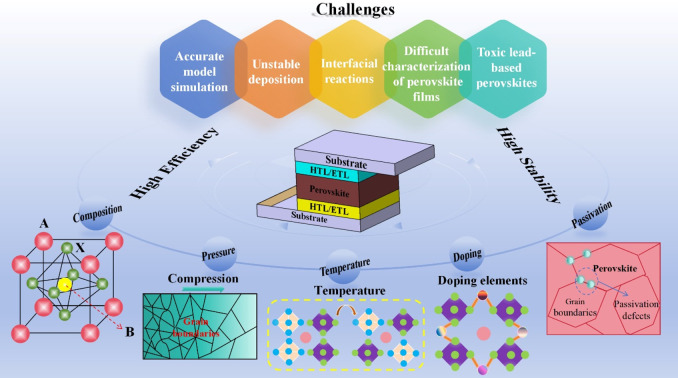

## 1. Introduction

**Fig. 1:**
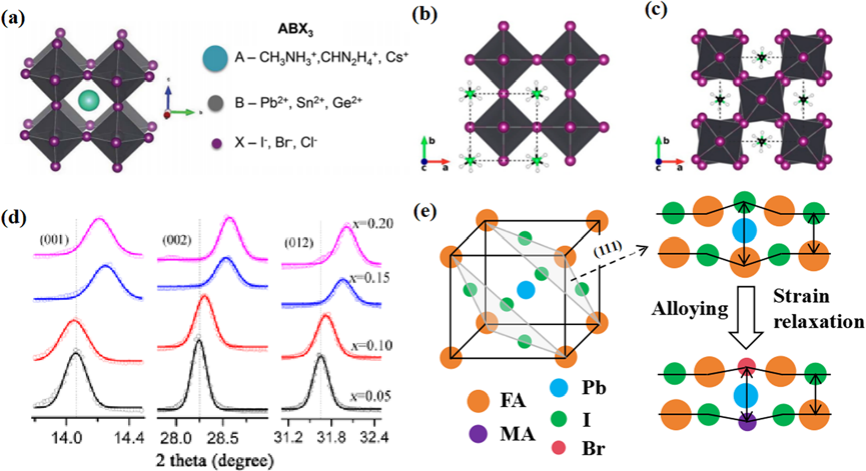
(a) Crystal structure of perovskite. Adapted with permission from ref. 1. Copyright 2015 John Wiley and Sons. Schematic structural representation of MAPbI_3_, where MA = methylammonium in the (b) pseudocubic and (c) tetragonal phases. Adapted with permission from ref. 4. Copyright 2020 American Chemical Society. (d) Dependence of powder X-ray diffraction (XRD) peaks on the value of *x* in (FAPbI_3_)_1−*x*_(MAPbBr_3_)_*x*_ single crystals. The hollow circles represent data points, and the solid lines are Gaussian fits of the data. The shift of all the peaks to higher 2*θ* implies contraction of the lattice due to alloying of FAPbI_3_ with MAPbBr_3_. Adapted with permission from ref. 2. Copyright 2019 American Chemical Society. (e) Schematic illustration of strain relaxation after MAPBr_3_ alloying into FAPbI_3_ (side view). Reprinted with permission from ref. 6. Copyright 2016 American Chemical Society.

Perovskite compounds with a chemical formula ABX_3_ have the three-dimensional (3D) crystal structure shown in Fig. 1a.^1^ The A-site cations are not thought to contribute directly to the band structure, but they play an important role in maintaining structural stability. This was achieved through charge compensation in the PbI_6_ octahedron, which is primarily facilitated by electrostatic (van der Waals) interactions with the inorganic cage.^2,3^ Methylammonium lead halide (MAPbI_3_) is a typical perovskite with a simple cubic lattice, in which Pb ions are centered on the octahedron and methylammonium (MA) ions are located between the octahedral building blocks. The most symmetric phase of MAPbI_3_ and its related materials is the cubic (*Pm3m*) lattice, which typically reduces symmetry through an octahedral tilting sequence transformation. The cumulative effect of these tilts leads to a phase transition from cubic to quadrilateral (Fig. 1b and c).^4^ Such transformations can alter the electronic band structure, consequently influencing the photoelectric properties of the material. The size of the A-site cations is a critical factor in this dynamic. Larger cations expand the lattice and reduce the band gap, while smaller cations have the opposite effect. The properties of PSCs can be tailored significantly *via* adjusting A-site cations. For example, by adding a cation with a small effective radius (MA^+^) to FAPbI_3_, the Goldschmidt tolerance factor can be adjusted to approximately 1.^5^ This has been achieved by either shrinking the lattice (Fig. 1d) or relaxing the crystal strain of the FA-based perovskite (Fig. 1e), thereby stabilizing the cubic phase of the perovskite.^6^ Much research has been dedicated to enhancing the performance of PSCs, with a primary focus on optimizing the perovskite composition, refining material preparation methods, innovating cell manufacturing processes, and exploring novel device structures.^7–9^ Despite the impressive performance of these devices, the intricate interactions between the perovskite layer and other functional layers can affect their formation and subsequent behavior.^10,11^

The quest for optimal PSC performance has intensified in recent years, with particular focus on the preparation of high-quality perovskite films and the reduction of charge-recombination losses within the perovskite layer and at the interfaces.^12,13^ In previous reports,^14–16^ perovskite microcrystals were mainly obtained by rapid nucleation crystallization in a saturated solution. However, the poor controllability of the nucleation process results in discontinuous microcrystalline films characterized by rough surfaces and large voids, which do not meet the uniformity and repeatability standards required for large-scale production.^17^ In addition, the organic hole transport layer (HTL) commonly used in solvent methods is expensive and exhibits poor stability,^18,19^ which leads to difficulties in subsequent electrode deposition and device manufacturing. Therefore, exploring deposition technologies suitable for industrial production has become a crucial focus in PSC research. Vapor-deposition methods are a promising solution for large-scale production as they enable the low-cost deposition of stable inorganic HTLs by using vacuum-deposition principles.^20^ Magnetron sputtering is one of the fastest developing vapor-deposition technologies in the electronics industry and has the advantages of a wide selection of suitable materials, uniform and dense film formation, and fast deposition.^21^ Peng *et al.* prepared fluorine-doped tin oxide (FTO)/NiO_*x*_/perovskite/SnO_2_/Ag devices by magnetron sputtering and achieved the magnetron-sputtering deposition of all the functional layers of a PSC for the first time.^22^ Both magnetron sputtering of the perovskite layer and continuous deposition on the perovskite layer destroy the soft lattice of the perovskite.^23^ A breakthrough in high-quality perovskite deposition *via* magnetron sputtering was achieved by post-treatment with methyl amine gas.^23,24^ Furthermore, to prevent potential damage caused by further deposition of SnO_2_, a mixed sputtering buffer layer of polymethacrylate (PMMA) and phenyl-C_61_-butyric acid methyl ester (PCBM) was deposited on the perovskite layer.^22^ This film protects the delicate lattice structure of the perovskite and ensures efficient charge transfer within the final cell.

**Fig. 2:**
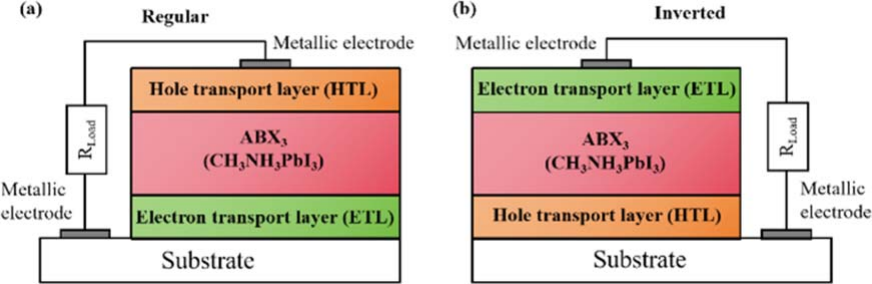
Basic structure of PSCs in (a) regular and (b) inverted configurations. Reprinted with permission from ref. 32. Copyright 2018 John Wiley and Sons.

Widely used conventional solution-based deposition methods present challenges related to solvent compatibility and thermal budget constraints,^25,26^ which limit the range of materials and structures that can be effectively applied to devices. Notably, it is challenging to deposit most metal oxides on top of perovskite layers using traditional solvent methods.^27^ An alternative approach involves the formation of stacked perovskite films *via* hot pressing,^28^ which effectively avoids solvent compatibility and thermal budget limitations. Lamination processes have emerged as an attractive method for manufacturing PSCs owing to their self-encapsulating nature and compatibility with high-throughput manufacturing methods.^29^ Furthermore, the pressure applied during processing can enhance the PSC performance by improving the interfacial microstructure between layers.^30,31^Fig. 2a illustrates the most successful and widely studied configuration, while Fig. 2b introduces an inverted structure.^11^ Regardless of the specific configuration, MAPbI_3_ is the most typical perovskite for photovoltaic applications. Its superior properties and performance make it a focal point of ongoing efforts to increase the efficiency and stability of PSCs to record levels.

Numerous studies have shown that polycrystalline perovskite films with large grains have a long carrier diffusion length, high mobility, and high photoluminescence quantum efficiency owing to their high crystal quality, high degree of orientation, low resistance, few grain boundaries, and low number of defects.^33,34^ However, the loss of organic cations or halide anions during thermal annealing can produce point defects, such as uncoordinated lead ions or halide vacancies. These defects can accelerate PSC degradation during operation under light irradiation and photothermal effects.^35^ In addition to thermal annealing, the accumulation of ions at the interfaces can initiate slow electrochemical reactions, potentially compromising the selective contact materials and triggering severe degradation.^36^ Even if the material is properly encapsulated (or measured under laboratory conditions in an inert atmospheres), the device may be unstable. In particular, electric field-induced ion transport can lead to chemical reactions with external iodide ions.^37,38^

This review focuses on the strategies for enhancing the performance of PSCs. First, the solvent-free preparation methods for perovskite materials and new techniques for the self-assembly of PSCs are discussed. The factors that degrade the power conversion efficiency (PCE) and stability of PSCs, current techniques employed to address these challenges, and potential avenues for further improvement are explored. This review summarizes the internal and external degradation mechanisms at the device and module levels by analyzing solvent-free methods for preparing perovskite films, PSC self-assembly methods, and the contact between the layers. Furthermore, existing problems and their potential solutions are proposed. This review provides a new idea for improving the PCE and module stability of PSCs.

## 2. Preparation of perovskite active layer by magnetron sputtering

Currently, the predominant deposition technology for perovskite active layer is the solvent method, which is widely recognized as a cost-effective manufacturing process that achieves devices with high PCE.^13,39^ However, commercial production is difficult with the solvent method as the film uniformity and repeatability are limited at large scales.^40^ The inherent difficulty arises from the uncontrollable volatility and fluidity of solutions. Solvents with excessively high or low volatility can lead to low perovskite film quality, resulting in poor photoelectric performance.^41^ In the solvent method, solvent evaporation inevitably results in the formation of numerous pinholes, which compromise the integrity and stability of the porous films.^42^ Furthermore, the residual dimethyl sulfoxide and amorphous phase at the interface of the perovskite film produce harmful cavities after irradiation, resulting in many nonradiative composite defects.^43^ In addition, the toxicity of the anti-solvent that needs to be added during the preparation of perovskite precursors is a serious problem.^44^ More information about the preparation of PSCs using various solvent methods can be found in previous reports.^44,45^ In contrast, there are many solvent-free methods to prepare perovskite layers, such as vapor deposition, molten salt method, solid phase method and mechanical synthesis. Among them, magnetron sputtering technology is a mature, established, and reproducible method for depositing thin films that is applicable at both laboratory and industrial scales.^46^ Besides, at present, there are few researches on the preparation of perovskite active layer by solvent-free method. At present, magnetron sputtering of perovskite active layer can reduce the direct contact between human and perovskite and improve the safety. Therefore, the use of magnetron sputtering for all functional layers can promote the commercialization of PSCs.^22^ Perovskite films prepared by magnetron sputtering exhibit enhanced properties, and PSCs fabricated on FTO-coated glass surfaces have promising commercial and industrial applications.^47,48^

**Fig. 3:**
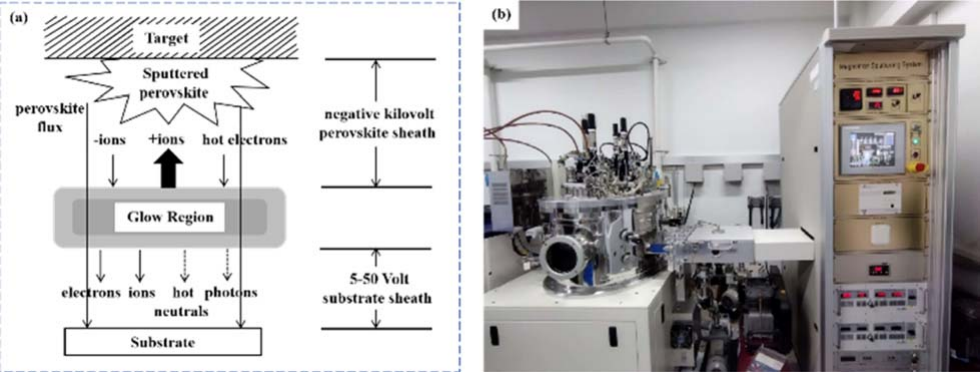
(a) Film sputtering process. (b) Picture of 4-inch sputtering equipment.

Another important issue is transitioning high-PCE PSC technology from laboratory-scale preparation to commercial-scale high-throughput production with minimal PCE loss. Magnetron sputtering technology has the characteristics of low cost, high object utilization, solvent-free preparation, and easy control. It can be used for large-scale and large-area production and is suitable for current commercial and industrial applications.^23^ Depending on the power supply frequency, magnetron sputtering methods are classified into direct current (DC) sputtering and radio frequency (RF) sputtering. During DC sputtering, the substrate is connected to the negative electrode as the cathode, and the anode is connected to the sputtering material. When RF sputtering, the substrate is usually connected to the ground, and the sputtering material is connected to the high-frequency power supply.^49^ The sputtering of perovskite films consists of four steps, as shown in Fig. 3a. (1) Ions are formed in the plasma and directed towards the perovskite target. (2) Ion-sputtering of the perovskite target occurs. (3) The ejected atoms are transported to the substrate. (4) The atoms condense and form a film on the substrate. The impact of atoms or ions sputters the surface due to the exchange of momentum between the high-energy material and atoms within the cathode target.^50^ Compared to thermal evaporation films, sputtered films have a higher density, smaller particle size, better adhesion, and exhibit overall properties closer to the characteristics of bulk materials. The choice between DC and RF sputtering usually depends on the requirements of the particular application and the desired film properties. Fig. 3b shows a sputtering system that can achieve both DC and RF sputtering.

**Fig. 4:**
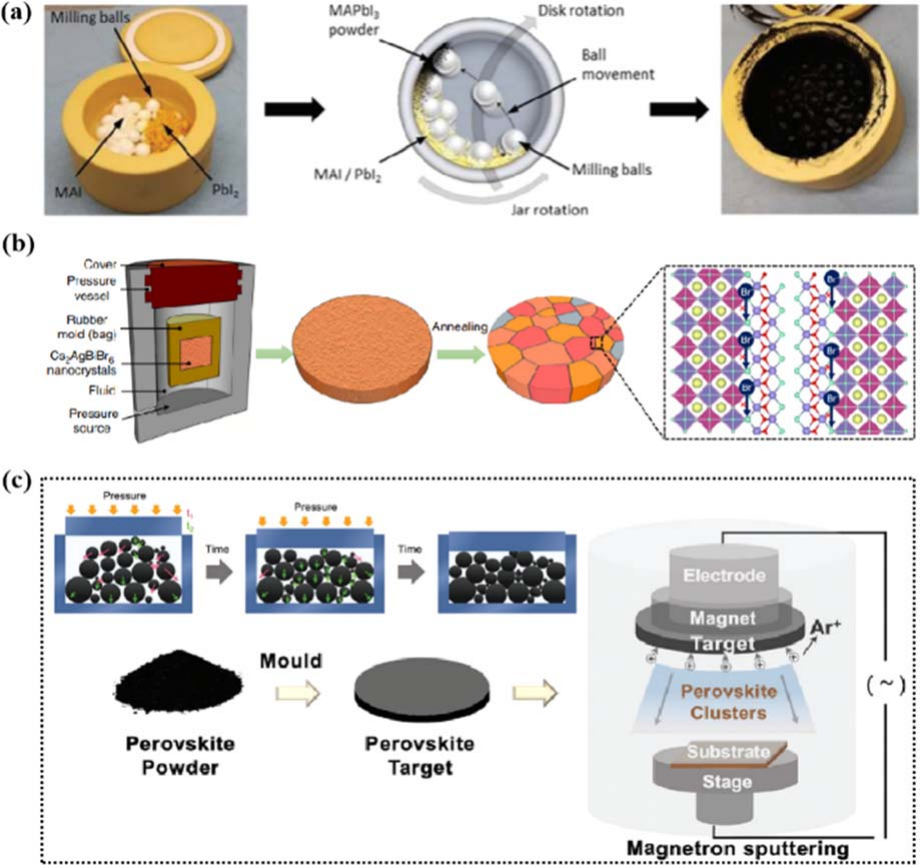
(a) Schematic diagram of the mechanochemical synthesis of perovskite powder by ball milling. Adapted with permission from ref. 53. Copyright 2019 American Chemical Society. (b) The schemes of isostatic pressure process and the compaction kinetics of perovskite powder during the pressing process. Adapted with permission from ref. 59. Copyright 2019 Springer Nature. (c) The perovskite layer preparation process *via* sputtering.^23,52^ Adapted with permission from ref. 23. Copyright 2021 John Wiley and Sons and ref. 53. Copyright 2019 American Chemical Society.

Magnetron sputtering was used to prepare perovskite films to form a high-quality active layer of high-performance polyvinyl chloride (Fig. 4).^51^ Perovskite materials can be effectively prepared by mechanical synthesis.^52,53^ In the straightforward mechanochemical method of producing halide perovskites, individual precursor powders, such as methylammonium iodide (MAI) and PbI_2_, are ground together with a mortar and pestle until the perovskite is formed, which is usually indicated by color changes.^23^ Finer and more controlled processes involve the use of a ball mill, such as a shaker or planetary ball mill.^54^ The reactant powder is weighed to the desired stoichiometry and added to the grinding vessel along with grinding balls, commonly made of stainless steel or toughened zirconia.^55^ Occasionally, a liquid abrasive (*e.g.*, cyclohexane) is added for wet ball milling (liquid-assisted grinding).^53,56^ The grinding tank is sealed tightly, inserted into the mill, and the synthesis process begins under the mechanical crushing by the balls.^55^ The mill pulverizes the reactants, thereby providing reaction energy through shock and friction.^57^ At longer timescales, the plastic deformation dynamics of the powder particles accelerate and initially dominate.^58^ The mechanically synthesized perovskite powder is subsequently molded and pressed into a target of a specific shape. For example, Cs_2_AgBiBr_6_ powder was initially formed into a cake and then subjected to a pressure treatment at 200 MPa using a hydraulic press.^23^ Additional annealing treatment further enhanced the crystallinity and grain growth. The perovskite target was installed in a magnetron sputtering instrument and sputtered onto a substrate to prepare the perovskite film. During sputtering, Ar^+^ ions bombard the target material to form perovskite clusters. Finally, a high-quality perovskite film was obtained.

**Fig. 5:**
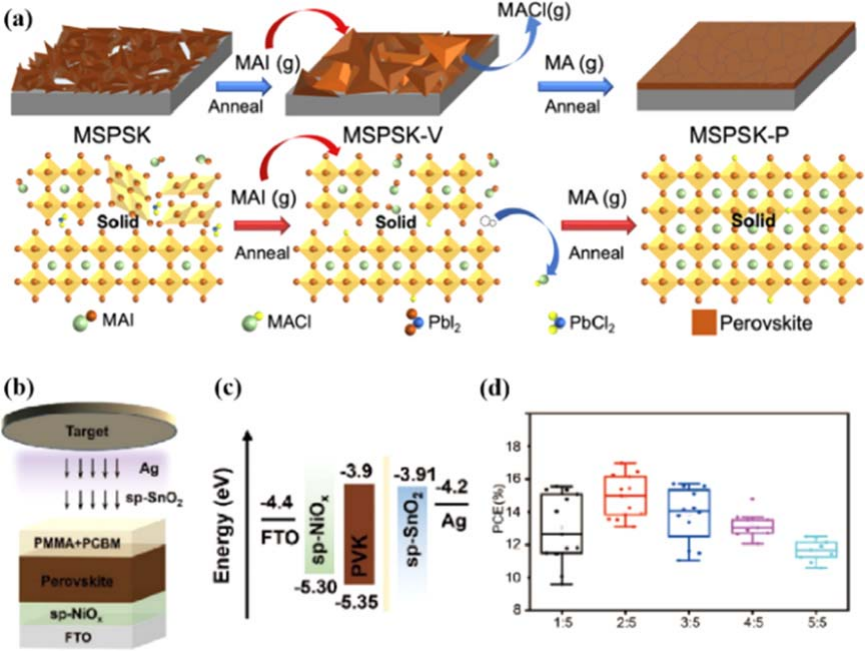
(a) Crystal growth mechanism of perovskite film during preparation. Adapted with permission from ref. 24. Copyright 2022 American Chemical Society. (b) Schematic of the device structure and fabrication process. (c) Energy band structure of the devices. (d) PCE statistics for sputtering buffer layers with different PMMA : PCBM ratios. Adapted with permission from ref. 22. Copyright 2023 American Chemical Society.

Solvent-free techniques have been investigated to enhance the PCE of PSCs.^60–63^ For instance, perovskite films have been deposited directly on mesoporous TiO_2_ substrates by sputtering MAPbI_3_ from a single target.^23^ Through post-treatment involving Cl doping and MAI/MA gas treatment, the defect density was reduced, leading to a successful increase in the PCE from 15% to 17.1%.^24^ Liu *et al.* first demonstrated a solvent-free method for preparing perovskite thin films for high-PCE PSCs *via* vapor deposition, in which the perovskite precursors were thermally evaporated in a vacuum chamber and deposited on the substrate.^60^ Furthermore, a laminated chemical vapor deposition technique for mixed-cationic PSCs incorporating self-passivation and gradient absorption layers was demonstrated.^39^ Magnetron sputtering enables mass production suitable for current commercial and industrial applications, and is commonly used to deposit the HTL and electron transport layer (ETL) of PSCs.^64,65^ Sputtering has also been proposed for perovskite film preparation, although device applications remain limited.^21,66^ Considering their specific properties, the other two most distinctive perovskites are the hybrid halides CH_3_NH_3_PbI_3−*x*_Cl_*x*_ and HC(NH_2_)_2_PbI_3_ composed of formamide (FA) cations.^67^ The incorporation of chlorine into CH_3_NH_3_PbI_3−*x*_Cl_*x*_ enhances the carrier transfer at the heterojunction,^68^ whereas FAPbI_3_-based devices exhibit broader infrared absorption.^69^ The perovskite materials were converted from powders to films *via* magnetron sputtering and post-treatment (MAI and MA gas vapor-assisted treatment).^23^ Notably, this process is beneficial for manufacturing tandem solar cells with a PCE improvement of nearly 60% relative to PSCs without post-processing. As shown in Fig. 5a,^24^ in contrast with the use of the pure MAPbI_3_ phase, PbCl_2_ in the perovskite film prepared by magnetron sputtering reacted with MAI during the gas-assisted treatment process, resulting in the successful introduction of Cl atoms to form MAPbI_3−*x*_Cl_*x*_. The transformation from a solid perovskite film (CH_3_NH_3_PbI_3−*x*_Cl_*x*_(s)) to a liquid perovskite film (CH_3_NH_3_PbI_3−*x*_Cl_*x*_·*x*CH_3_NH_2_(l)) occurs during MA gas introduction, resembling the dissolution of stacked solid perovskite nanoparticles into a continuous liquid phase.^24,70^ The subsequent removal of MA gas transforms the liquid perovskite film back into a solid form (formula (1)), resulting in a compact and smooth film with few pinhole defects. This Cl introduction process enabled the PCE of the PSC to be increased to 17.1%.^24^

1 CH_3_NH_3_PbI_3−*x*_Cl_*x*_ (s) + *x*CH_3_NH_2_ (g) ↔ CH_3_NH_3_PbI_3−*x*_Cl_*x*_·*x*CH_3_NH_2_ (l)

Because magnetron sputtering operates based on the principle of momentum exchange, a wide range of materials can be fabricated, such as various metals and oxide ceramics.^24,71^ All functional layers of a PSC can be prepared by magnetron sputtering.^23,46,72,73^ Replacing the organic charge-transport layer with a sputtered inorganic layer both reduces the cost and improves stability. However, subsequent deposition on the perovskite layer can damage the soft lattice structure of perovskite materials. To address this issue, a protective layer of PMMA and PCBM was deposited on the top of the perovskite layer by spinning coating to prevent further sputtering charge transport layer from damaging the perovskite layer (Fig. 5b–d).^22^ The protective layer prevents the perovskite from being damaged by subsequent SnO_2_ deposition while maintaining efficient charge transfer, resulting in a PCE increase from 14.62% to 17.43%.^22^ These findings demonstrate the considerable potential of magnetron sputtering in the preparation of all functional layers in PSCs. This innovative preparation process opens up new avenues for the industrialization of PSCs.

**Fig. 6:**
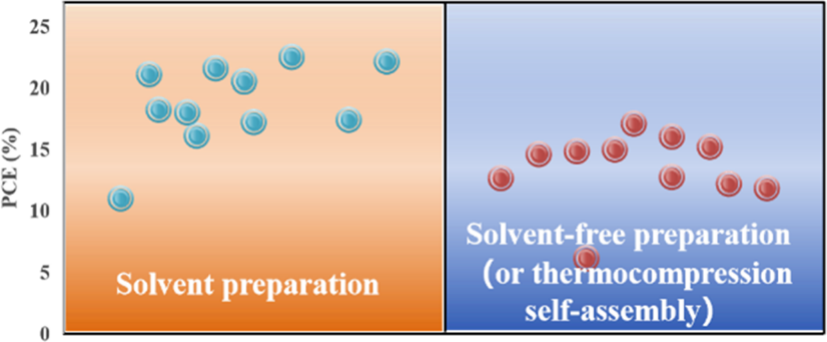
Comparison of efficiency between traditional solvent method and solvent-free method or thermocompression self-assembly for preparing PSCs.^22,23,28,33,76–80^

Applying an excessively high voltage during the sputtering process results in larger sputtering particles, leading to the formation of a rough film. Conversely, an excessively low voltage slows the sputtering process, causing perovskite decomposition.^74^ In addition, the thickness of the perovskite film plays a crucial role in device performance and can be controlled by adjusting the sputtering time.^23^ It was proposed that the optimal conditions for forming ∼400–500 nm perovskite layers were as follows: an applied voltage of 800 V, pressure of 9.6 Pa, and sputtering time of 5 min, conducted in an atmosphere with less than 30% humidity or in a nitrogen environment.^23^ To avoid the emergence of pre-liquids and improve the performance of PSCs prepared using solvent-free methods, many researchers have explored alternative processing methods. For example, a solid-state in-plane growth method was presented, which involves applying pressure and heat to grow two-dimensional (2D) layers on top of 3D layers, ensuring the consistent performance of the layers across the device.^75^ This approach successfully avoids the formation of unexpected phases, and allows the thickness of the 2D layer to be adjusted. The controllability of the 2D layer thickness facilitates device optimization to increase the built-in potential at the junction, which resulted in a stable PCE of 24.35% and high stability.^75^ However, the overall operational process is intricate, costly to control, and not readily scalable for large-scale production. Fig. 6 compares the efficiencies of PSCs prepared by solvent- and solvent-free methods (or thermocompression self-assembly), and it can be seen that the PCE of PSCs prepared by the solvent method is generally higher than that of PSCs prepared by the solvent-free method (or thermocompression self-assembly).^22,23,28,33,76–80^ Consequently, more efforts are required to find a cost-effective, scalable solvent-free method for preparing high-performance PSCs.

## 3. Perovskite surface defects and general improvement strategies

In high-quality perovskite films, surface defects are the main factor affecting the performance of PSCs, especially Pb vacancies, I vacancies, and Pb–I inverses.^81^ Various approaches have been employed to mitigate these surface defects, such as adding additives to the precursor solution, anti-solvent treatment, and post-treatment. These strategies introduce compounds that form coordination or ionic bonds with the charged defects within the perovskite structure.^24,82^ These passivating agents neutralize charges at the defect sites and reduce the disruptive effect of electrons on the ionic lattice, thereby affecting the structure of the perovskite layer.^83^ These agents also significantly affect film formation, further altering the volume defect concentration and terminating the perovskite surface. Effective interface management by post-processing is essential for improving both device PCE and stability. Many functional molecules have been used to passivate Pb-based defects and inhibit ion migration through the formation of coordination/ionic bonds.^39,84–86^ To study the control of Pb-based defects, the effect of amphoteric 2-methylthio-2-imidazoline (MT-Im) cations on the formation of interface defects of a new low-dimensional perovskite (LDP) was reported.^87^ The defect-forming energy of perovskites containing MT-Im was calculated using density functional theory (DFT).^88^ Compared with the formation energy of neutral defects on the perovskite surface,^89^ the effect of MT-Im on the formation of Pb–I anti-position defects in the perovskite was significantly higher than that of the control. This indicates that MT-Im has a pronounced passivation effect on the Pb-based defects in the 3D perovskite films.

**Fig. 7:**
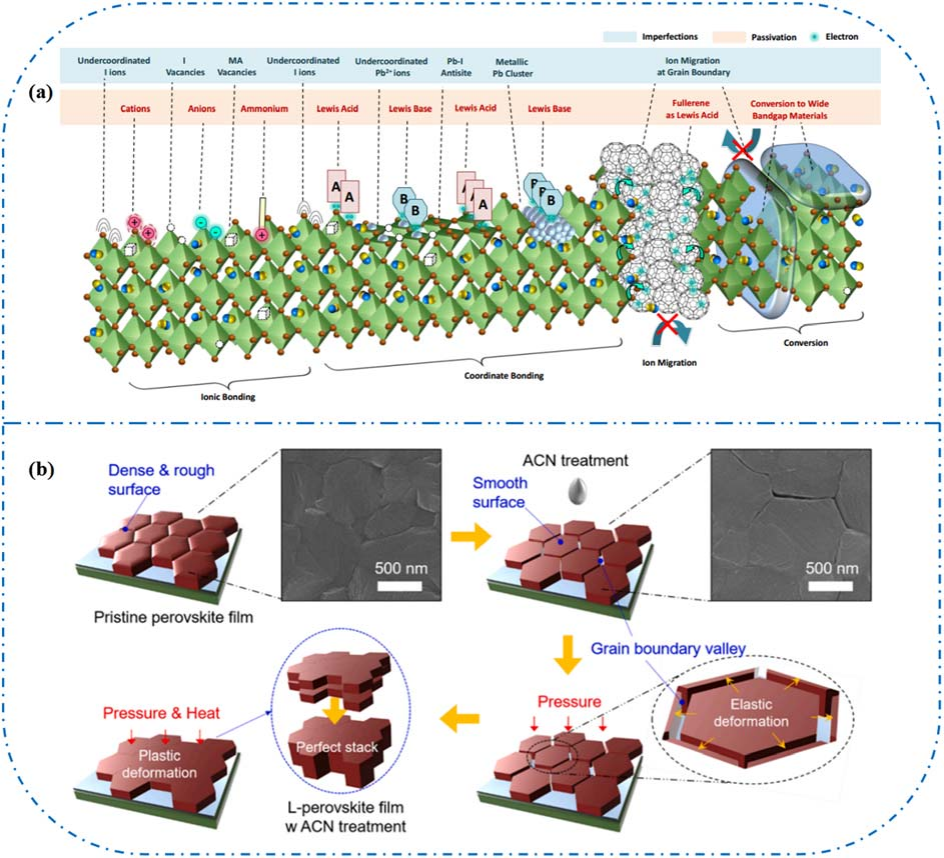
(a) Common surface defects for perovskites and the effect of various additives. Adapted with permission from ref. 90. Copyright 2020 John Wiley and Sons. (b) Schematic diagram of ACN-treated lamination behavior of layer-perovskite films during hot pressing. Adapted with permission from ref. 76. Copyright 2022 Elsevier.

Fig. 7a shows a schematic of the effects of common surface defects and additives on perovskite films.^90^ Major defects at the surface or grain boundaries of perovskite crystals can lead to deep traps, such as incongruous halide ions or Pb^2+^, and Pb clusters, and occasionally some inherent point defects caused by certain growth or processing conditions, such as Pb–I anti-position defects (PbI_3_).^91^ The surface of a control perovskite film was rough with an average grain size of 595 nm, as shown in Fig. 7b.^76^ After treatment, the smooth surface can reduce the probability of local contact between the top and bottom perovskite films, thus preventing the formation of large void defects. Moreover, the wide grain boundary valley facilitated the elastic deformation of the perovskite films under applied pressure in the maximum strain range of 5% (the elastic modulus of the perovskite films was 10–20 GPa).^30^ Subsequently, the plastic deformation of the perovskite film was caused by heat transfer from top to bottom, yielding an optimally layered perovskite film through a hot-pressing process. After annealing, all perovskite films had large grains and maintained a uniform, pinhole-free, and flat morphology. Notably, the perovskite films produced by hot pressing sublimation (HPS) treatment had larger single-layer particles.^33^ The appearance of sufficient PbI_2_ units near or at the grain boundaries results in the formation of PbI_2_ clusters, which act as barriers that impede further expansion of the grain boundaries, thereby inhibiting perovskite crystal growth.^92^ Prolonged or high-temperature thermal annealing leads to the loss of chloride ions through the formation of CH_3_NH_3_Cl (MACl), which leaches from the perovskite lattice, and the formation of PbI_2_, which significantly affects the long-term stability of PSCs. Conversely, excessive MAI results in a strong release of MACl, leading to poor-quality perovskite films with large pinholes, subsequently reducing the overall cell performance.^93^ In formula (2), MAI replaces most of the MACl at the grain boundaries, allowing the majority of MACl to escape from the perovskite film.^33^ The active MAI vapor and exchange reaction between MAI and MACl activate the grain boundaries, promoting the rapid migration of grain boundaries and grain growth. The optimal orientation and large grain size effectively inhibited volume and/or surface recombination of the perovskite films.^12,94^2
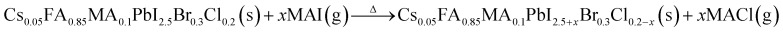


**Fig. 8:**
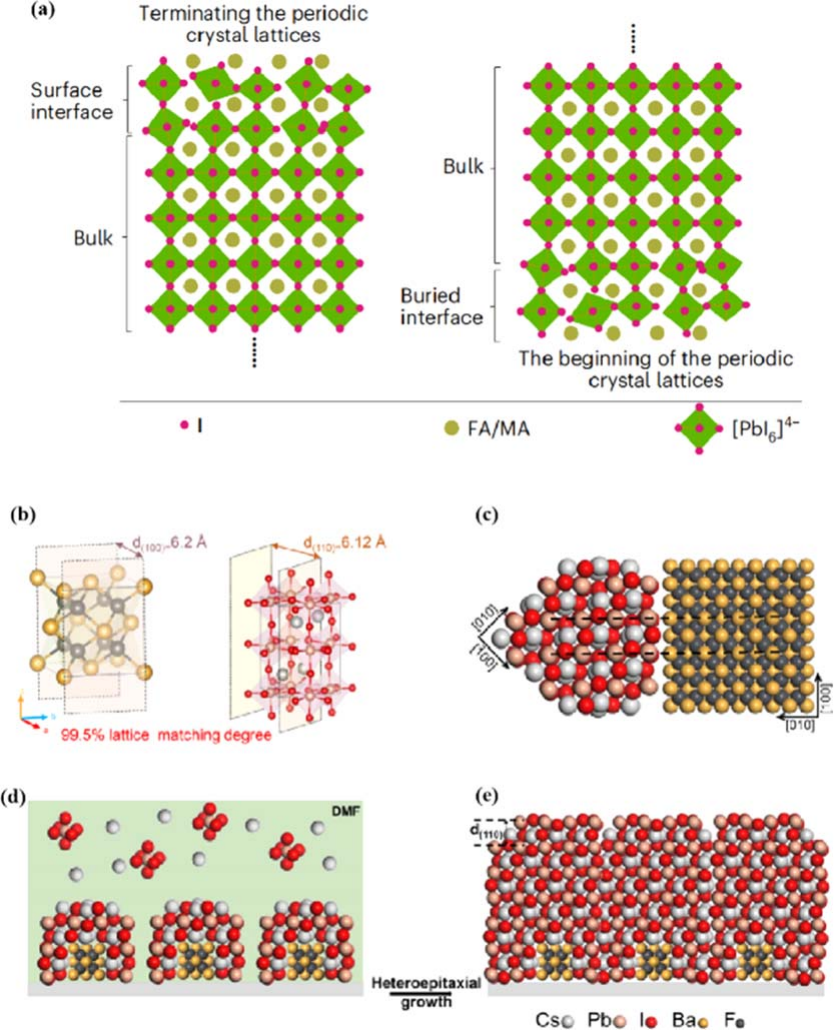
(a) Termination and beginning of the periodic crystal lattice. Adapted with permission from ref. 43. Copyright 2023 Springer Nature. (b) The unit cell structure of α-BaF_2_ (left) and γ-CsPbI_3_ (right). These two planes have a 99.5% lattice matching degree. (c) Cross-section of lattice matched heterostructure between α-BaF_2_ and γ-CsPbI_3_. (d and e) Structural modeling of the heteroepitaxial growth of γ-CsPbI_3_ thin film on the α-BaF_2_ nanoparticle substrate. Adapted with permission from ref. 96. Copyright 2022 American Chemical Society.

The abovementioned methods focus on the treatment of the exposed upper surface of the perovskite because this is easier to modify than the hidden lower surface.^12,76,80^ However, optimizing the lower perovskite surface is very important. Owing to the growth direction of the perovskite from bottom to top, the periodic structure must be terminated, and the perovskite lattice inevitably dissolves the upper surface of the perovskite film in the range of tens of nanometers, resulting in many defects and the formation of amorphous phases (Fig. 8a).^95^ The surface of the underlying ETL is used as the starting point for 3D periodic perovskite lattice epitaxial growth, which can be alleviated.^96^ Potassium chlorobenzene sulfonate was used as a new multifunctional agent to modify the buried tin oxide (SnO_2_)/perovskite interface of conventional polyvinyl chloride.^97^ The enhanced carbon–chlorine bonds effectively interacted with the uncoordinated Sn atoms, effectively filling the oxygen vacancies on the SnO_2_ surface. The synergistic effect of functional-group-rich organic anions and potassium ions can reduce the defect density, carrier recombination, and hysteresis. A high PCE of 24.27% was achieved for the improved device. A SnO_2_ ETL treated with formamidine sulfonic acid (FSA) ions helped rearrange the stacking direction, orientation, and distribution of the residual PbI_2_ in the perovskite layer, thereby reducing deleterious side effects of residual PbI_2_.^98^ FSA functionalization also modified the SnO_2_ ETL to suppress deep defects at the perovskite/SnO_2_ interface.

In addition to a high defect density, the buried interface also has problems related to residual strain, low crystallinity, excessive ion migration, and harmful voids.^43,99^ Research on passivating agents has not yet found a comprehensive solution to these problems. The construction of benign buried interfaces remains an ongoing challenge, particularly when passivation-free approaches are used. γ-CsPbI_3_ solid-solution epitaxial growth without strain was achieved using an α-BaF_2_ nanoparticle substrate.^96^ The prepared γ-CsPbI_3_ thin films were uniform and smooth. The lattice match between the highly exposed surface on the α-BaF_2_ nanoscale heteroepitaxy growth substrate and the γ-CsPbI_3_ (110) surface was 99.5% (Fig. 8b–e). A transparent and conductive perovskite (SrSnO_3_) was demonstrated as an ETL.^43^ A high degree of lattice matching makes the growth of halide perovskites on the ETL more orderly, avoiding the deterioration of the buried interface. The constructed buried interface exhibited limited defects and strain, better crystallinity, reduced ion migration, and fewer lattice growth inevitably results in poor-quality lower and upper interfaces of the perovskite films, respectively. By utilizing ETL layers that better match the perovskite lattice and surface treatment of the upper surface of the perovskite, poor initiation and fewer terminations of amorphous phases can be achieved, resulting in more efficient PSCs.

## 4. Device performances

Due to the low cost, solution processability, and unprecedented rapid improvement in photovoltaic performance, PSCs are currently the focus of next-generation photovoltaic research.^66,100^Table 1 summarizes the properties of various functional layer materials constituting PSCs and their respective devices. Functional-layer materials such as ETLs/HTLs exhibit minimal alterations, primarily achieved by perovskite component engineering, surface engineering treatment of functional layers, or improving processing technology to optimize PSC performances. The solar cells composed of perovskite active layers commonly used in the past five years are selected, and each solar cell was treated by different methods. By comparing the PCE of each device, it is found that the efficiency of the device prepared by solvent-free method and hot-pressing self-assembly is generally lower than that of the traditional method. Therefore, although the preparation of solar cells by solvent-free method or hot-pressed self-assembly can reduce the high cost and solution toxicity caused by traditional methods, the improvement of its PCE needs to be further improved.

**Table 1:** Different functional layers and properties of PSCs^a^

TCO	ETL	Perovskite absorber layer	HTL	Electrode layer	Efficiency	Stability	Year (Ref.)
FTO	SnO_2_	MAPbI_3_	NiO_*x*_	Ag	14.62% (magnetron sputtering all layers)	93.5% (nitrogen box for 2000 h)	2023 (ref. 22)
FTO	SnO_*x*_	CH_3_NH_3_PbI_3_, MAPI	NiO_*x*_	No	10.6% (two half stacks)	—	2018 (ref. 101)
FTO	TiO_2_	MAPbI_3_ + PbCl_2_	Spiro-OMeTAD	Au	17.10% (magnetron sputtering perovskite)	85% (after 1000 h of storage)	2022 (ref. 24)
FTO	SnO_2_	2D (BA)_2_PbI_4_ and 3D (FAPbI_3_)_0.95_(MAPbBr_3_)_0.05_	PTAA	Au	24.35%	94% after 1056 h under the damp heat test (85 °C/85% relative humidity) and 98% after 1620 h under full-sun illumination	2021 (ref. 75)
FTO	TiO_2_	FA_0.90_MA_0.05_Cs_0.05_PbI_2.85_Br_0.15_	Spiro-OMeTAD	Au	22.52%	96% after 2000 h	2022 (ref. 76)
FTO	PTAA	FACsPbI_3_	Spiro-OMeTAD	Au	22.06%	92.9% after 1000 h of aging	2021 (ref. 84)
FTO	TiO_2_	Cs_0.10_FA_0.75_ MA_0.15_Pb(I_0.85_Br_0.15_)_3_	Phthalocyanine	Au	Over 20%	Little decrease in the PCE after 1000 h at 85 °C	2021 (ref. 102)
FTO	SnO_2_	Mixed-halide perovskites	Spiro-OMeTAD	Au	11.70%	80% after 30 h under illumination	2022 (ref. 103)
FTO	SnO_2_	Cs_0.05_PbI_2.05−*x*_Cl_*x*_	Spiro-OMeTA/PTAA hybrid	Au and Ag	24.42%	Negligible decline in performance after storage in dry air for more than 4000 h	2022 (ref. 104)
FTO	TiO_2_	MAPbI_3_	Spiro-OMeTAD	Au	15.22% (magnetron sputtering perovskite)	80% (stored under nitrogen atmosphere and at a temperature of ≈15–25 °C for 1000 h)	2021 (ref. 23)
FTO	SnO_2_	Inactive (PbI_2_)_2_RbCl stabilizes FAPbI_3_	Spiro-OMeTAD	Au	25.6%	96% after 1000 h of shelf storage and 80% after 500 h of thermal stability testing at 85 °C	2022 (ref. 105)
FTO	SnO_2_	Cs_0.05_FA_0.85_MA_0.1_Pb(I_0.9_Br_0.1_)_3_	Spiro-OMeTAD	Au	22.15%	96% after aging for 2520 h under ambient conditions and 87% after light irradiation for 1000 h	2021 (ref. 33)
ITO	SnO_2_	Cs_0.05_(FA_0.77_MA_0.23_)_0.95_Pb(I_0.77_Br_0.23_)_3_	PTAA	No	17.24% (two half cells)	∼90% in the 85 °C shelf test for over 3000 h, ∼95% under AM 1.5 G	2023 (ref. 28)
ITO	SnO_2_	(Cs_0.03_FA_0.97_PbI_3_)_0.95_(MAPbBr_3_)_0.05_	Spiro-OMeTAD	Au	24.07%	92% after 4200 h under 45 ± 5% RH	2021 (ref. 87)
ITO	MeO-2PACZ	Rb_0.05_Cs_0.05_MA_0.05_FA_0.85_Pb(I_0.95_Br_0.05_)_3_	C_60_	Ag	Over 25%	87% after over 2400 h of 1-sun operation at about 55 °C in air	2022 (ref. 106)
ITO	2PACz	2D Cs_0.03_(FA_0.90_MA_0.10_)_0.97_PbI_3_	C_60_/BCP	Ag	24.3%	>95% after >1000 h at heat	2022 (ref. 16)
ITO	SnO_2_	(FAPbI_3_)_1−*x*_MAPb(Br_3−*y*_Cl_*y*_)_*x*_	Spiro-OMeTAD	Au	15.27%	—	2021 (ref. 107)

a TCO – transparent conductive oxide

Understanding the degradation mechanisms caused by various factors is crucial for developing strategies for improving device performance. Degradation factors are typically classified as internal/inherent factors arising from the internal structural and chemical instability, or external factors stemming from the external environment.^2^ Inherent degradation factors primarily originate from the material itself and the interfaces between functional layers. The mechanisms of degradation caused by external factors need to be better understood to reduce the influence of internal factors on degradation. To enhance the performance of PSCs, it is essential to understand the degradation mechanisms induced by external factors, given the interactions between external and internal degradation factors.^108^ External degradation factors such as the water and oxygen concentrations play a significant role. Water molecules break hydrogen bonds within the perovskite, forming new hydrogen bonds and hydration compounds, leading to high ion mobility and severe water degradation.^1^ Ions in perovskite can react with oxygen to form charge barriers and electron traps, while oxygen can create Pb–O bonds on the surface of perovskite films, resulting in severe oxygen degradation.^109^ Encapsulation partially or completely eliminates degradation caused by water and oxygen. However, external degradation factors such as light and heat, which are challenging to isolate, remain persistent threats to device stability. Understanding and addressing these factors are crucial for enhancing the performance of PSC devices. Overall, internal factors relate to the intrinsic properties of the material and structure, whereas external factors are related to the conditions under which a solar cell is used during its practical application. Optimizing the devices to minimize the effects of the degradation factors can improve the performance and stability of PSCs.

**Fig. 9:**
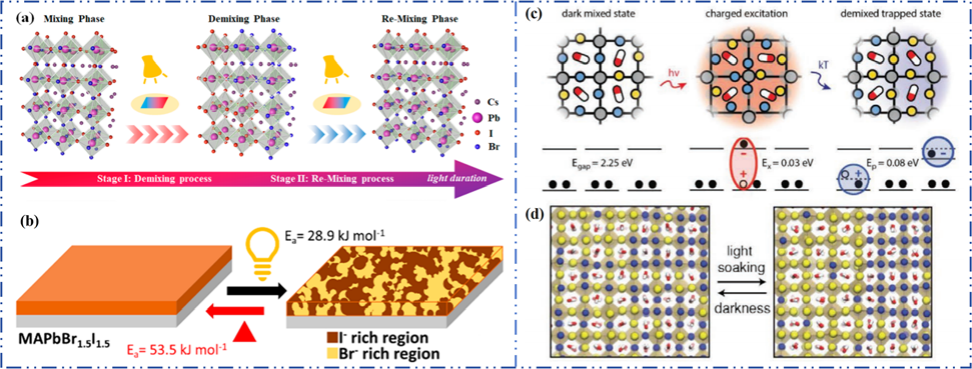
(a) 3D schematic diagram of the photoinduced structure change. Adapted with permission from ref. 111. Copyright 2021 American Chemical Society. (b) Reversibility of photoinduced halide segregation. Adapted with permission from ref. 112. Copyright 2020 American Chemical Society. (c) Schematic of photoinduced polaron trapping and associated energy levels. (d) Schematic of macroscale phase segregation with same color key. Adapted with permission from ref. 90. Copyright 2020 John Wiley and Sons.

Light exposure can induce ion migration, trap-state formation, phase separation, and photocatalytic degradation, significantly affecting the practical applications of PSCs.^110^ The photoinduced self-healing process involves two main stages: demixing in stage I and remixing in stage II. Fig. 9a shows the photoinduced self-healing process through I^−^/Br^−^ ion displacement under continuous illumination.^111^ Additionally, Fig. 9b shows the reversibility of the photoinduced halide segregation, that is, the photoinduced phase segregation and subsequent recovery under dark conditions.^112^ Compositional changes that occur naturally before light exposure produce I-rich regions with reduced band gaps. Upon irradiation, electron–hole pairs rapidly separate, with carriers moving to low-gap I-rich regions before recombination. In these regions, a substantial number of carriers interact with the high-valence perovskite structure, causing lattice deformation through electron–phonon coupling (Fig. 9c).^90^ This strain was sufficient to increase the enthalpy of mixing, resulting in a second minimum in the free energy and Br content plot, resulting in the perovskite forming an I/Br-rich phase (Fig. 9d).^90^ Continual light exposure causes most of the light-excited charges to migrate toward the I-rich, low-gap regions on the film, where they are extracted and subsequently recombined. Owing to the smaller gap, these carriers produced less voltage than those from the stoichiometric mixed-halide region. However, when the light is removed, the free energy diagram of the perovskite returns to its pre-illumination state, reestablishing the single-phase driving force.^113^ Therefore, phase segregation is typically reversible displacement under continuous illumination.^111^ Additionally, Fig. 9b shows the reversibility of the photoinduced halide segregation, that is, the photoinduced phase segregation and subsequent recovery under dark conditions.^112^ Compositional changes that occur naturally before light exposure produce I-rich regions with reduced band gaps. Upon irradiation, electron–hole pairs rapidly separate, with carriers moving to low-gap I-rich regions before recombination. In these regions, a substantial number of carriers interact with the high-valence perovskite structure, causing lattice deformation through electron–phonon coupling (Fig. 9c).^90^ This strain was sufficient to increase the enthalpy of mixing, resulting in a second minimum in the free energy and Br content plot, resulting in the perovskite forming an I/Br-rich phase (Fig. 9d).^90^ Continual light exposure causes most of the light-excited charges to migrate toward the I-rich, low-gap regions on the film, where they are extracted and subsequently recombined. Owing to the smaller gap, these carriers produced less voltage than those from the stoichiometric mixed-halide region. However, when the light is removed, the free energy diagram of the perovskite returns to its pre-illumination state, reestablishing the single-phase driving force.^113^ Therefore, phase segregation is typically reversible.

**Fig. 10:**
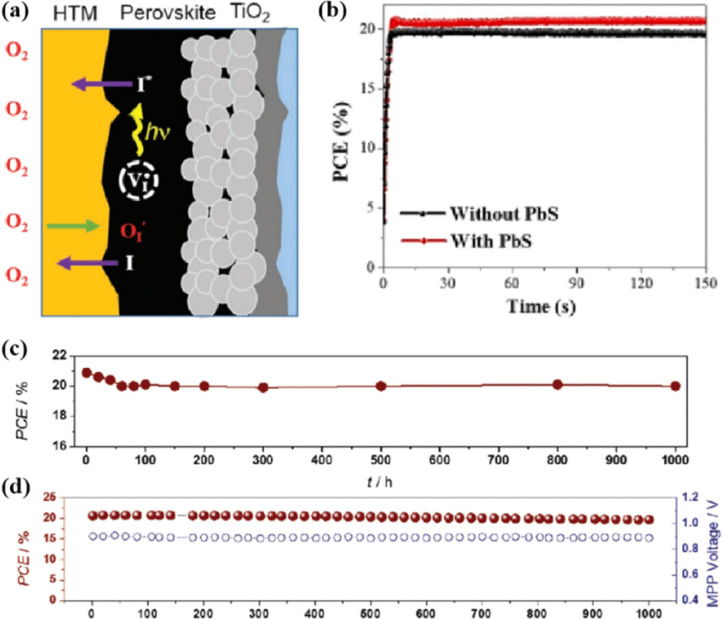
(a) Schematic to describe the activation of iodine diffusion by oxygen and light. Adapted with permission from ref. 117. Copyright 2019 John Wiley and Sons. (b) Maximum power point tracking (MPPT) results for devices. Adapted with permission from ref. 80. Copyright 2023 Elsevier. (c) Shelf test results of the encapsulated device under 85 °C and 85% RH conditions and (d) long-term operational stability of the encapsulated devices under simulated real operating conditions (maximum power point tracking under 1 sun AM 1.5 G illumination including UV without controlling temperature in ambient air). Adapted with permission from ref. 117. Copyright 2019 John Wiley and Sons.

In addition, the migration of atoms/ions from organometalide perovskites into the HTL induces degradation, affecting both the PCE and stability of the PSCs.^114^ Oxygen plays a key role in the photoinduced degradation of PSCs, in which iodine diffuses from the perovskite to the HTL under the influence of O_2_ and light. Photoexcited holes combine with iodine ions, forming neutral iodine with high activity that may diffuse into the HTL, where the iodine activity is significantly lower.^115^ In addition, experimental results have shown that O_2_ provides enhanced iodine activity and generates iodine vacancies in the perovskite, thereby enhancing the diffusion of iodine outside the perovskite (Fig. 10a).^116,117^ Iodide migration involves charge transport from the perovskite to the HTL. Therefore, to solve the problem of photoinduced degradation in an oxygenated atmosphere, it is necessary to inhibit the diffusion of iodine or isolate the device from oxygen. A solid buffer layer of PbS deposited on the surface of perovskite effectively increases device stability by preventing perovskite degradation.^80^ The PbS buffer layer prevented direct contact between the perovskite and water and inhibited ion migration in the buffer layer, resulting in a PSC with significantly improved long-term stability under high humidity, high temperature, and continual illumination conditions.^80,118^ Furthermore, the PbS buffer layer enhanced the extraction of holes from the perovskite, thereby improving the power conversion efficiency (Fig. 10b).^80^ Similarly, a layer of np-Al_2_O_3_ was deposited between the perovskite and HTL as a diffusion barrier, resulting in a device with good stability against oxygen and light exposure.^117^ The PSC devices were encapsulated by sealing the surface of the top layer of the device with adhesive sheets, thereby removing the air trapped during lamination. Shelf tests were performed at 85 °C and 85% relative humidity (RH) to assess the package efficiency (Fig. 10c). After 1000 h, the PCE of the device remained at 96% of its initial value (20.0–20.9%). Long-term stability tests under actual operating conditions revealed a PCE of 20.6% (96% retention of its initial value; Fig. 10d), highlighting the robustness of the device packaging.

Heat is another inevitable external factor that introduces phase transitions and hinders separation, decomposition, ion diffusion, and charge transport.^119^ The operating temperature of solar cells can rise to 85 °C, while the phase transition of the most commonly used perovskite material (MAPbI_3_) usually occurs at 60 °C.^119^ The liquid crystalline nature of perovskites facilitates the movement of ions.^120^ Recent findings have demonstrated that the thermal activation of two different colloidal solutions or physically paired MAPbBr_3_ and MAPbI_3_ films results in the rapid exchange of halide ions.^121–123^ The difference in spectral characteristics between bromide and iodide perovskite films makes it possible to track the movement of halide ions. The rate of mixing (or homogenization) increases with increasing temperature, indicating that a thermally activated process determines the mixing and exchange of halide ions.^124–129^ While the migration rate of halide ions is slow at room temperature, it increases significantly at elevated temperatures, leading to an accelerated rate of dark reduction.^4^ The mixing entropy explains the thermally activated mixing of halide ions to produce mixed-halide perovskites.^124,130,131^

**Fig. 11:**
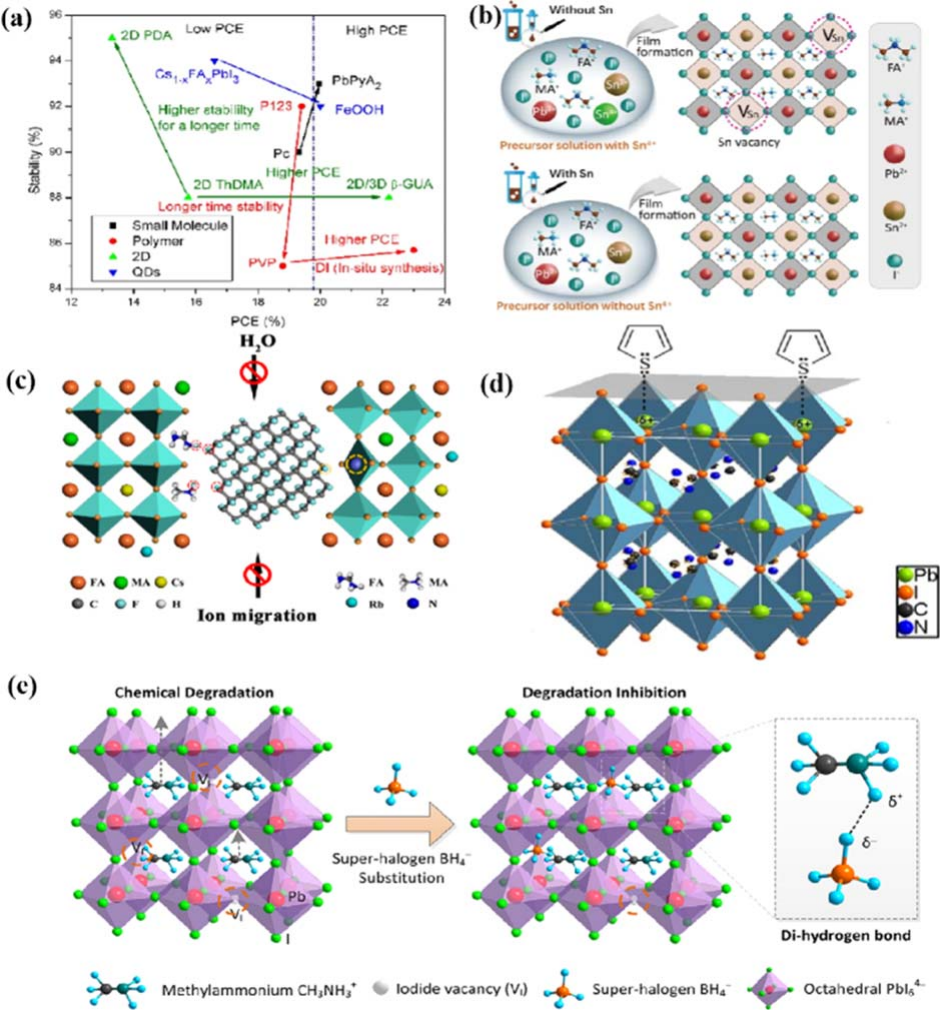
(a) The relationship between the light stability and PCE of PSCs after interface modification of different perovskite layers. Adapted with permission from ref. 108. Copyright 2021 American Chemical Society. (b) These results indicate that the presence of Sn^4+^ in the precursor solution leads to the formation of Sn vacancy in the mixed lead–tin perovskite, and the absence of Sn^4+^ inhibits the formation of Sn vacancy in tin-reduced precursor perovskite. Adapted with permission from ref. 134. Copyright 2019 Springer Nature. (c) Schematic diagram of the active role of stripped fluorographene quantum dots as passivating agents in perovskite films. Adapted with permission from ref. 48. Copyright 2020 American Chemical Society. (d) Passivation of undercoordinated Pb^2+^ on perovskite surface by thiophene or pyridine. Adapted with permission from ref. 91. Copyright 2019 Royal Society of Chemistry. (e) The superhalogen BH^4−^ aggregates at the iodide ion vacancy, forming a hydrogen bond with methyl ammonium (CH_3_NH_3_^+^). Adapted with permission from ref. 78. Copyright 2020 American Chemical Society.

Chi *et al.* introduced a method for graphing the stability as a function of PCE, by employing a quantitative analysis to provide insights for material selection, development, and structural design to achieve a balance between PCE and stability (Fig. 11a).^108^ There are two fundamental strategies for enhancing the performance of PSCs: component and interfacial engineering. Composite material engineering is realized through the material development of each main layer and metal electrode.^132^ Although the formation of a uniform solid solution is not always guaranteed, certain combinations of A-site cations and B-site halides in perovskites are superior to their single-cation/halide counterparts in terms of both PCE and stability.^2^ Interfacial engineering further improves device performance by passivating the primary layer to reduce defects and/or inserting a protective layer around the fragile perovskite absorption layer and the ETL to mitigate degradation factors.^133^Fig. 11b–e illustrate the various improvement strategies. However, achieving both high PCE and stability is a major challenge. Specifically, it is difficult to quantify stability while enhancing the PCE. This stems primarily from the absence of standardized aging measurement conditions and criteria. Perovskite absorbers, which are the main source of device degradation owing to their inherent instability, significantly influence the device PCE and stability.^108^

### 4.1. PCE

MAPbI_3_ usually undergoes a phase transition from the tetragonal to cubic phase at temperatures of 54–57 °C.^135^ Organic MA cation volatilization occurs at higher temperatures because of the weak bonding and low formation energy. The thermal degradation of perovskite materials can be explained by the Goldschmidt tolerance factor, which is an empirical indicator reflecting the stability of perovskite crystals based on the ionic radius within the crystal lattice.^136^ Perovskite materials exhibit good stability when the tolerance factor is in the range of 0.8–1.0. Deviations from this range lead to severe thermal degradation due to lattice distortions.^137^ Many efforts have been made to improve the coverage and quality of perovskite films, such as the optimization of the precursor solution, deposition conditions, and post-treatment. For instance, additives like PbCl_2_ are used to regulate the crystallization kinetics of perovskite films, resulting in better film coverage, reduced defects, and increased carrier diffusion length.^138–143^ The combination of large FA and small Cs is beneficial for the formation of the preferred black perovskite phase, removal of volatile substances, and improvement of thermal stability.^108^ Therefore, substituting MA with Cs and FA is an efficient approach for fabricating thermally stable PSCs. By adjusting the composition of the A and X sites, the thermal stability of low- or high-PCE PSCs can be significantly improved.^144^ Owing to the same electron configuration, Sn is a good substitute for Pb; however, Sn^2+^ is easily oxidized to Sn^4+^, which greatly reduces the stability of the perovskite.^134^ The incorporation of Pb and Sn helps to reduce the oxidation of Sn^2+^, promote the transformation of PbI_2_ into a perovskite phase, diminish grain boundaries, enhance crystal quality, increase the open-circuit voltage (*V*_oc_), and improve the overall PCE and stability. For example, a MAPb_0.9_Sn_0.1_I_3_ PSC device, in which Sn partially replaced Pb, maintained 90% of its original PCE of 18.3% after 500 h of illumination.^77^ Moreover, the introduction of Br into Sn-based PSCs further improves their stability owing to the reduction in Sn vacancies.

**Fig. 12:**
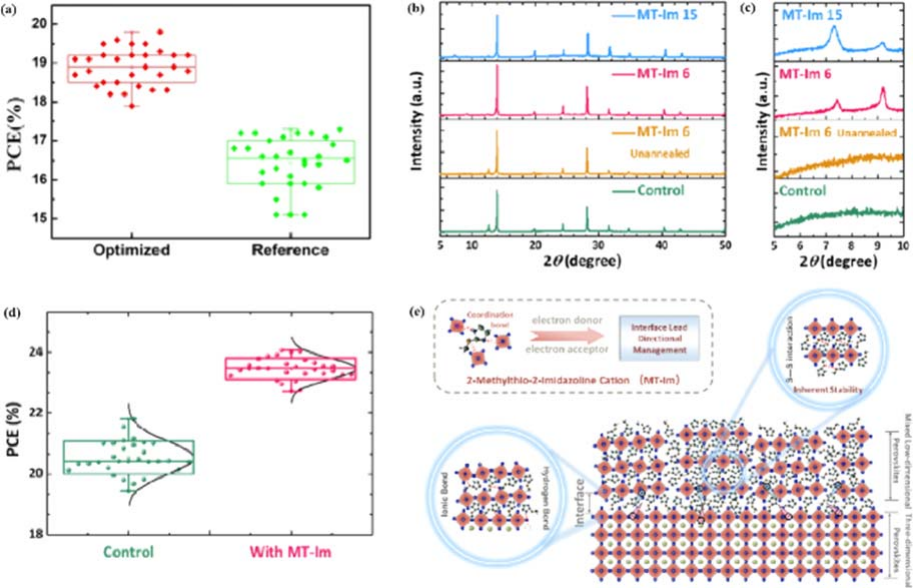
Box charts of (a) PCE of optimized TEPA-MAPbI_3−*x*_Cl_*x*_ and reference MAPbI_3−*x*_Cl_*x*_-based devices. Adapted with permission from ref. 139. Copyright 2020 American Chemical Society. (b and c) XRD patterns of the control, unannealed MT-Im (6 mg mL^−1^), and MT-Im (15 mg mL^−1^) perovskite films. (d) PCE distributions for the control and MT-Im perovskite devices. (e) Schematic illustration of the effective management by inherently stable MT-Im LDP for the Pb-based defects at the interface. Adapted with permission from ref. 87. Copyright 2020 American Chemical Society.

Although the PCE of PSCs has improved rapidly, long-term stability problems persist under different conditions. 2D PSCs have the advantage of high stability based on interfacial cations alternating between amino and halogen ions; however, 2D PSCs exhibit low PCEs compared to 3D devices.^145^ This discrepancy arises from the limitations imposed by the 2D structure on the efficient transmission of principal charges. This issue was addressed by incorporating a linear short chain of tetraethylene pentamine (TEPA) into a perovskite structure to modify its symmetry.^139^ Devices based on TEPA-MAPbI_3−*x*_Cl_*x*_ had a higher PCE compared to the original PSC based on MAPbI_3−*x*_Cl_*x*_ (Fig. 12a).^139^ Furthermore, a heterocycloammonium salt cation (2-methylthio-2-imidazoline; MT-Im) LDP passivation layer was added to a perovskite film.^87^ The growth of the mixed-phase LDP introduced a strong interaction with under-coordinated Pb^2+^ on the surface of the perovskite film. Although the addition of MT-Im did not alter the crystal structure of the perovskite (Fig. 12b),^87^ two new diffraction peaks appeared, indicating the formation of mixed-phase layers on the bulk perovskite surface.^47,146^ These peaks corresponded to the (MT-Im)_2_PbI_4_ and (MT-Im)PbI_3_ mixed-phase layers (Fig. 12c). The PCE of the improved device increased by 24.07% (Fig. 12d). As shown in Fig. 12e, as electron donors, C=N and –S–CH_3_ with electron lone pairs in MT-Im exhibit strong Lewis acid–base interactions with unpaired Pb^2+^, forming Pb–N and Pb–S coordination bonds.^147^ Nitrogen also interacts with –NH_3_^+^ in perovskites to form hydrogen bonds. These synergistic effects result in a robust passivation effect.^130,131^ This prevents fewer non-radiative composite centers and enhances the connection between the surface LDP and the massive perovskite, facilitating the separation and transfer of internal electrons and holes.

### 4.2. Stability

**Fig. 13:**
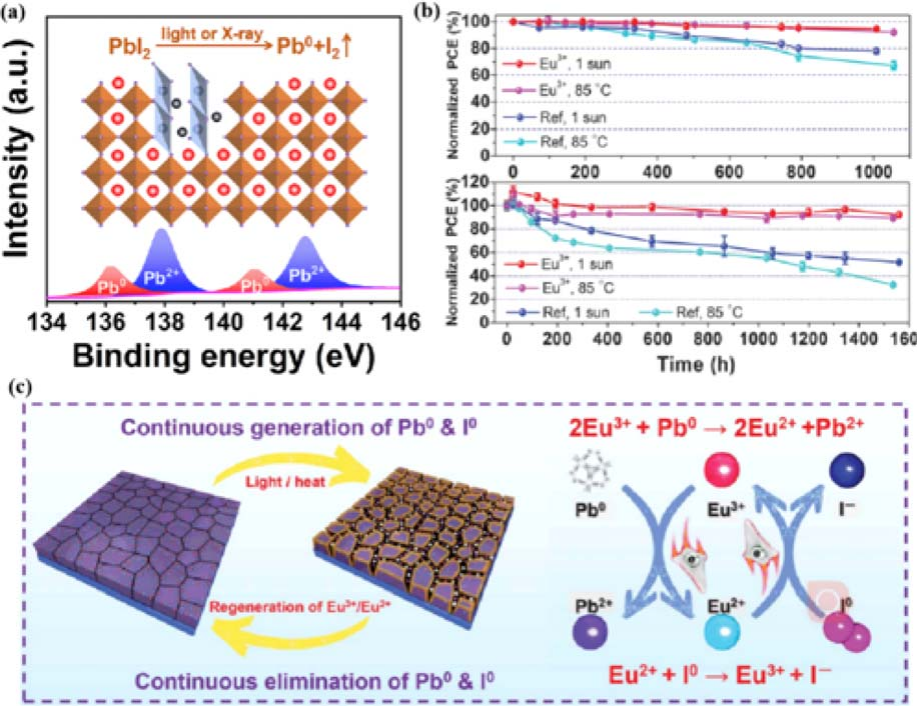
(a) Pb^0^ source diagram. Adapted with permission from ref. 17. Copyright 2022 Elsevier. (b) The PCE evolution of Eu^3+^–Eu^2+^-incorporated and reference devices under 1 sun illumination or 85 °C aging condition. (c) Proposed mechanism diagram of cyclically elimination of Pb^0^ and I^0^ defects and regeneration of Eu^3+^–Eu^2+^ metal ion pair. Adapted with permission from ref. 159. Copyright 2019 The American Association for the Advancement of Science.

The PCEs of PSCs have been successfully improved by additive engineering and surface passivation. However, a critical challenge is the poor stability of halide perovskites, which poses a significant obstacle to their imminent commercialization.^148–151^ The stability limitations of PSCs are classified into external and internal factors. External environmental factors can be prevented by using external packaging. Intrinsic factors such as photoinduced decomposition, ion migration, and thermal degradation^152^ must be addressed to improve the quality of perovskites. The PCE and stability of PSCs are influenced by the presence of defects, such as the Pb–I inverse and unpaired metallic lead (Pb^0^) at the interface.^87^ Pb^0^ is considered one of the most harmful intrinsic factors leading to the deterioration of PSC performance.^153–156^ For high-performance PSCs, Pb^0^ caused by the thermal annealing of perovskite films or degradation of the interface between the perovskite and ETL should be avoided.^38,157^ It was found that Pb^0^ defects are a byproduct of the decomposition of residual PbI_2_ in the perovskite under light or X-ray irradiation (Fig. 13a).^17^ In contrast, perovskites without excess lead halides demonstrated a reduced propensity to form Pb^0^ impurities, exhibiting better tolerance to light and X-ray exposure. The presence of Pb^0^ hindered perovskite crystallization, increased deep-layer defect levels, reduced trap activity, enhanced nonradiative recombination, and accelerated perovskite degradation.^38,158^ Therefore, the PCE and stability of PSCs obtained from photoaged lead-iodide films containing Pb^0^ impurities were significantly reduced. Furthermore, a europium ion pair (Eu^3+^–Eu^2+^) was used as a redox shuttle to selectively oxidize Pb^0^ and reduce I^0^ defects simultaneously during cyclic phase transitions.^159^ The synthesized device achieved a high PCE while maintaining long-term stability (Fig. 13b). Eu^3+^ can easily be reduced to Eu^2+^ with a stable half-full f7 electron configuration, forming naturally bound ion pairs.^12^ The redox shuttle facilitated the transfer of electrons from Pb^0^ to the I^0^ defect through a cyclic process, where Eu^3+^ oxidizes Pb^0^ to Pb^2+^, and the resulting Eu^2+^ simultaneously reduced I^0^ to I^−^ (Fig. 13c). The reaction between Pb^0^ and I^0^ is thermodynamically favorable, with a standard molar Gibbs formation energy of 173.6 kJ mol^−1^ for PbI_2_(s),^159^ which provides the driving force for the elimination of these two defects. The proposed redox shuttle eliminated the corresponding defects based on the following chemical reactions:^159^

3 2Eu^3+^ + Pb^0^ → 2Eu^2+^ + Pb^2+^

4 Eu^2+^ + I^0^ → Eu^3+^ + I^−^

The stability of PSCs is essential for meeting the requirements of industrial and commercial applications. However, their poor stability has become a major bottleneck restricting the comprehensive performance of PSCs and has thus attracted much research attention.^160^ Achieving a highly stable perovskite requires that the tolerance factor is kept within a range of 0.8–1 and preferably maintaining a cubic phase with a tolerance factor of 0.9–1. Tolerance factors can be modulated by changing or mixing ions of different sizes, which offers a promising avenue for enhancing stability.^2^ The introduction of mixed cations promotes the growth of crystals with preferred orientations and the formation of smooth perovskite films. This reduces the number of trap states, accelerates the extraction and transport of electrons, reduces charge recombination, and enhances thermal stability.^108^ Mixing halides is beneficial for reducing oxidation under light illumination, increasing the charge-transport rate, adjusting the bandgap, and consequently improving stability. The substitution of I^−^ with Cl^−^ ions at the X site promotes carrier transport and device stability. Cl^−^ at the X site facilitates the crystallization of MAPbI_3_, increases the diffusion length of carriers, accelerates charge transfer, and enhances carrier collection, thus improving efficiency and stability.^108^ PSCs are susceptible to inherent structural instabilities associated with the presence of inorganic halide anions and organic cation vacancies, resulting in the deterioration or even premature failure of devices. The partial substitution of I^−^ by BH^4−^ reduces the vacancy density, inhibits recombination, and forms a smooth, dense, and high-quality perovskite film, which enhances the PCE and stability.^78^ Br and I^−^ have similar ionic radii, and partially replacing I^−^ with Br results in a cubic rather than a tetragonal structure, reducing octahedral tilt and lattice distortion. Combining Br and I compresses the lattice, increases the binding strength of MA–Pb, increases the grain size, and improves the PCE and thermal stability.^161^ However, excessive Br at the X site can cause halide segregation, resulting in dephasing, the formation of I-rich inclusions that act as recombination centers, and poor PSC stability. Optimizing the Br concentration is crucial for obtaining stable PSCs with high PCE. Although ion substitution at the A and/or X sites plays a dominant role in improving performance, the substitution of Pb^2+^ at the B site contributes to enhanced photostability and thermal stability. This was attributed to high light absorption, long carrier lifetime, low charge-transport resistance, high carrier-recombination resistance, and low trap density.^78^

**Fig. 14:**
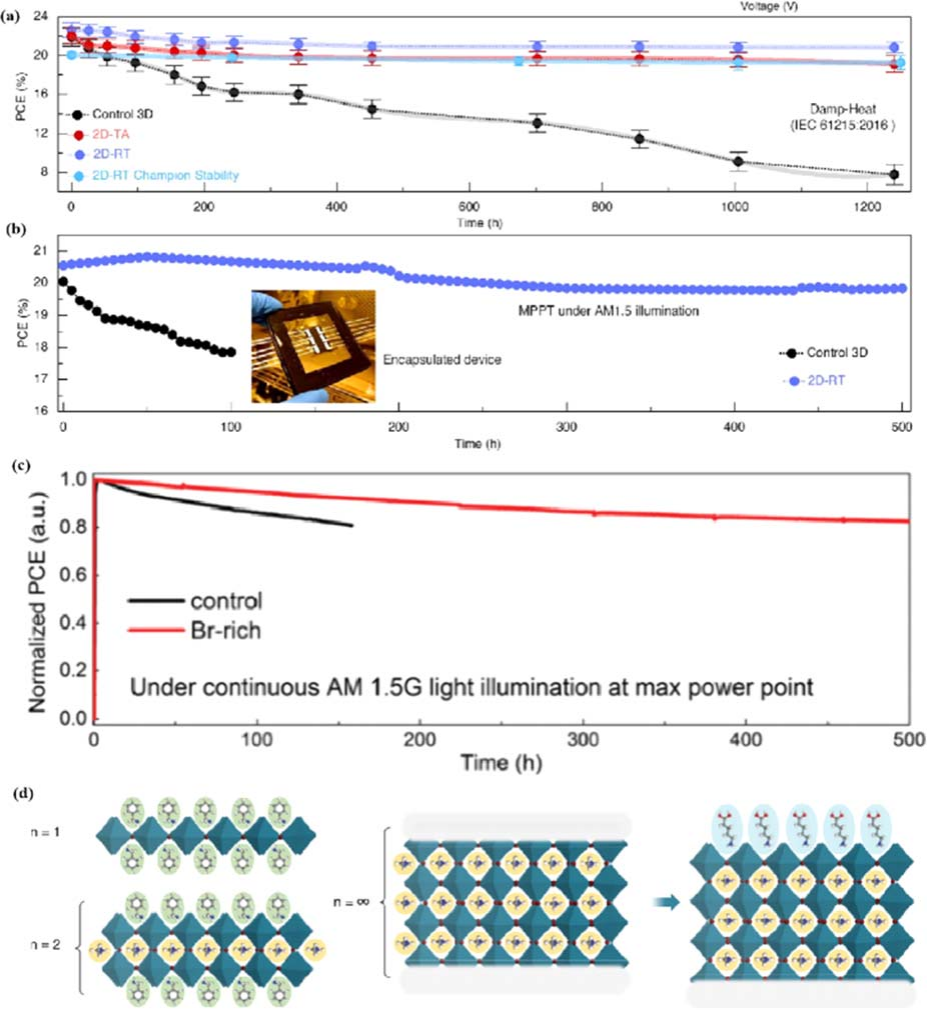
(a) Variation of the PCE at damp-heat test of encapsulated devices. (b) Continuous MPP tracking for the encapsulated control and 2D-RT cells under AM 1.5 illumination in ambient air. 2D-RT: 2D-perovskite layers when post-treatment was performed at room temperature. Adapted with permission from ref. 16. Copyright 2022 The American Association for the Advancement of Science. (c) Long-term stability test result for the PSCs fabricated with control and Br-rich perovskite film, conducted at MPP bias under continuous AM 1.5 G illumination. Adapted with permission from ref. 162. Copyright 2019 John Wiley and Sons. (d) Using large cations to adjust the dimension. Adapted with permission from ref. 167. Copyright 2018 The American Association for the Advancement of Science.

Compared to 3D perovskite devices, devices using low-dimensional perovskites, in which some or all cations are replaced by large organic ligands, show greater stability against heat, light, and moisture during long-term operation.^144,145^ For example, the development of damp-heat-stable PSCs involves tailoring the dimensional fragments of a 2D perovskite layer. This layer, formed by oleoamine-iodized molecules at room temperature, effectively passivated the perovskite surface at the electron-selective contact.^154^ The resulting inverted PSCs exhibited a PCE of 24.3% under wet-heat test conditions and maintained 95% of their initial value after 1000 h (Fig. 14a), thus meeting one of the key industrial stability criteria for photovoltaic modules.^16^ However, large ligands hinder charge transfer and reduce efficiency (Fig. 14b).^16^ It was found that after 500 h of continuous operation, the initial PCE of a Br-rich device remained above 80% (Fig. 14c).^162^ This device showed the best operational stability among PSCs prepared by sequential deposition methods and was even comparable to PSCs prepared using a one-step anti-solvent droplet method. This emphasizes the significance of the Br content in enhancing the operational stability of PSCs.^163^ The high bromine content stabilizes the perovskite phase of FAPbI_3_, making it thermodynamically favorable and achieving superior thermal stability.^164^ In addition, because iodide anions exhibit low ion migration activation energies, particularly under light, their easy migration during operational stability tests can degrade PSC device performance. Therefore, substituting bromine with iodine can enhance the operational stability of PSC devices. Alternatives such as short-chain cations^165^ and conjugated cations^166^ can potentially increase the PCE without sacrificing stability (Fig. 14d). By exploiting the hydrophobicity of large cations, the stability of perovskites can be effectively improved by adjusting their dimensions.^167^ Protecting the perovskite interfaces and grain boundaries is another way to slow degradation.

## 5. Thermocompression self-assembly PSCs and properties improvement strategies

**Fig. 15:**
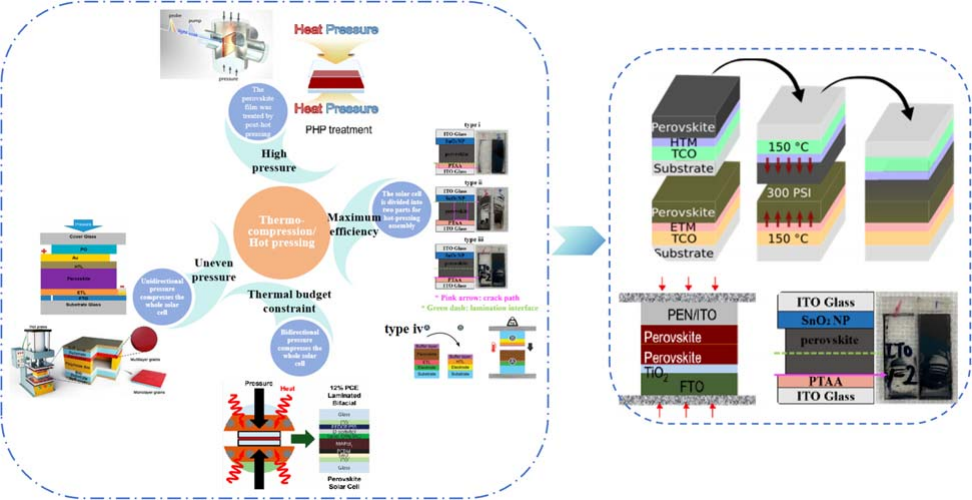
Different hot pressing methods. Adapted with permission from ref. 28. Copyright 2023 American Chemical Society. Adapted with permission from ref. 30. Copyright 2019 American Chemical Society. Adapted with permission from ref. 33. Copyright 2021 John Wiley and Sons. Adapted with permission from ref. 76. Copyright 2022 Elsevier. Adapted with permission from ref. 101. Copyright 2018 American Chemical Society. Adapted with permission from ref. 103. Copyright 2022 American Chemical Society. Adapted with permission from ref. 168. Copyright 2019 American Chemical Society. Adapted with permission from ref. 169. Copyright 2020 John Wiley and Sons.

Currently, the main methods for improving the quality of perovskite films are based on post-fabrication hot pressing or pressurization during PSC packaging. However, these methods introduce additional operational steps and require consideration of solvent compatibility and thermal budget constraints.^143^ Consequently, some researchers have proposed dividing PSCs into two separate devices for hot-press assembly as a potential solution to these challenges (Fig. 15).^28,30,33,76,101,103,168,169^ Notably, this approach resulted in three different interface types: HTL/perovskite, ETL/perovskite, and perovskite/perovskite. Jung *et al.* studied three interface forms formed by hot pressing.^28^ They observed that with the transformation of the perovskite surface into a block, the PSCs exhibited a buried interface solely between the perovskite layer and two functional transport layers. The hot-pressing process facilitated the development of larger perovskite grains as well as smoother and denser interfaces. This resulted in a reduction in the defect and trap densities and repressed ion migration and phase segregation under light exposure. Moreover, the hot-pressed perovskite layers demonstrated enhanced water resistance. The conventional layer-by-layer solution treatment of lead–halide perovskite devices imposes limitations on their structures. The layer beneath the perovskite must withstand the strong organic solvent used during perovskite formation, whereas the layer above operates within a limited thermal budget and must be treated with a nonpolar solvent to prevent perovskite degradation.^13^ Because the phase segregation of mixed-halide perovskites involves the migration of halide ions. The crystal defects and strain lattices at the grain boundaries allow active ions to migrate from the grain boundaries, thereby accelerating halide segregation.^127^

Even in single-halide perovskite systems such as MAPbI_3_, ion migration has been identified as a primary driver of degradation.^126,170,171^ Both single-halide and mixed-halide perovskites can benefit from an increase in the activation energy of ion migration, which effectively reduces the degradation rate and extend the operational lifetime of the device. As the pressure increased, the final mixing ratio of the separated phase approached that of the mixed phase, thereby altering the thermodynamic pattern of phase segregation.^172^ In addition, it was found that phase segregation was much slower at high pressures for all MAPb(Br_*x*_I_1−*x*_)_3_ mixing ratios (*x* = 0.25, 0.5, and 0.7).^170^ The enhanced activation energy of halide migration under a compressed unit-cell volume indicates an increased barrier to halide diffusion into vacancies under pressure.^114^ This suggests that a reduction in the unit-cell volume achieved through component engineering or physical pressure can effectively delay halide migration by increasing the activation barrier of the migration process. Lamination not only reduces the band gap of the perovskite, but also preserves or sharpens the characteristics of the absorption band edge, corresponding to changes in the structure and associated defects of the conduction and/or valence band edges.^173^ Additionally, reducing the grain boundary open space and limiting the content of mobile ions at the grain boundary can inhibit the migration of mobile grain-boundary defects, thereby inhibiting halide segregation.^103^

Currently, the main methods for perovskite film treatment involve hot pressing of either the perovskite films themselves or the entire PSC. In the former approach, a thin perovskite film is deposited onto a suitable substrate and subjected to hot pressing at high temperature (typically 100–200 °C) and pressure (usually 10–50 MPa).^76,174,175^ This helps improve the perovskite crystal structure and reduce defects. For instance, a metal halide perovskite (MHP) film was deposited on clean bare glass and hot-pressed at various temperatures and a pressure of 80 MPa (glass cannot withstand 100 MPa).^103^

By limiting the growth of grains in the plane direction, open-structure grain boundaries were transformed into dense GBs, thereby improving structural homogeneity. This inhibits defect-mediated ion migration at the grain boundaries, resulting in MHP solar cells with PCEs exceeding 11%.^103^ Full-device treatment involves heating the prepared PSC in a hot-press machine, thereby enhancing the interfacial contact between the perovskite film and the HTL and ETL. For example, a stack was placed in a hot press and annealed in a nitrogen-filled glove box at 150 °C and 2 MPa for 5 min, resulting in a PCE of 10.6%.^101^ It was found that the self-assembly of PSCs in two stacks increases the PCE while reducing the number of preparation steps.^28^ This innovative technique involves laminating two separately treated half-stacks, enabling the use of unique device architectures and greater flexibility in selecting the ETL and HTL materials.^169^ As mentioned earlier, the application of hot pressing during lamination can improve the crystal quality of perovskites, thereby reducing the defect density, leakage current, and iodine migration in the perovskite.^28^ The performance of PSCs prepared by hot pressing is highly dependent on the applied pressure and temperature, as summarized in Table 2. Additionally, factors such as doping of the perovskite or surface passivation significantly affect the performance. Therefore, a detailed analysis of the various influencing factors and an understanding of their mechanisms are crucial for improving PSC performance. The enhancement mechanisms employed in the solution preparation of PSCs should also apply to solvent-free processes.

**Table 2:** Process parameters of hot pressing encapsulated PSCs

Perovskite material	Temperature (°C)	Pressure	Time (min)	Hot pressing interface	Efficiency	Stability	Year (Ref.)
FA_0.90_MA_0.05_Cs_0.05_PbI_2.85_Br_0.15_	120	5.5 MPa	10	Perovskite–perovskite	22.52%	96% after 2000 h	2022 (ref. 76)
(FAPbI_3_)_1−*x*_MAPb(Br_3−*y*_Cl_*y*_)_*x*_	85	—	10	Only heat treated perovskite	16.50%	—	2021 (ref. 107)
Methylammonium lead iodide (MAPI)	150	300 PSI	20	Perovskite–perovskite	10.6%	—	2018 (ref. 101)
Cs_0.05_(FA_0.77_MA_0.23_)_0.95_Pb(I_0.77_Br_0.23_)_3_	180	7.5 MPa	10	Perovskite–perovskite	17.24%	∼90% in the 85 °C shelf test for over 3000 h, ∼95% under AM 1.5 G, 1-sun illumination in an ambient atmosphere for over 600 h	2023 (ref. 28)
Mixed-halide perovskites	150	80 MPa	30	Perovskite–glass	11.70%	80% after 30 h under illumination	2022 (ref. 103)
Cs_0.1_(MA_0.17_FA_0.83_)_0.9_Pb(I_0.83_Br_0.17_)_3_	90	50 MPa	5	Poly (triarylamine)–poly (triarylamine)	14.6%	87% after almost 100 h of maximum power point tracking	2020 (ref. 169)
Mixed-cation perovskite	150	2 MPa	15	Perovskite–polyimide	22.15%	96% after aging for 2520 h under ambient	2021 (ref. 33)
Mixed halide perovskite	65	7 MPa	10	Entire PSCs devices	13.67%	—	2020 (ref. 31)
CsPbI_2_Br	120	—	10	Only heat treated perovskite	16.40%	96% after heating at 85 °C for 1000 h	2020 (ref. 176)
Mixed perovskite Cs_0.05_(FA_0.85_MA_0.15_)_0.95_Pb(I_0.85_Br_0.15_)_3_ and (FAPbI_3_)_0.85_(MAPbBr_3_)_0.15_	Room temperature	400 mbar	5	Entire PSCs devices	Improved 7% compared to no pressure	—	2019 (ref. 30)

### 5.1. Composition-based enhancement

**Fig. 16:**
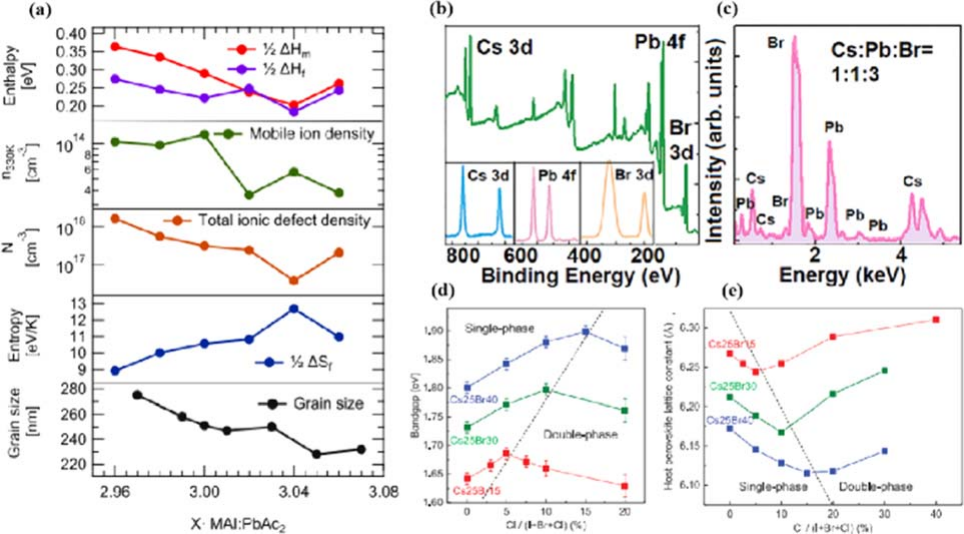
(a) Comparison of iodine-related defect properties in MAPbI_3_ to micro-structural characteristics as function of stoichiometry. Adapted with permission from ref. 175. Copyright 2022 American Chemical Society. (b) Surface X-ray photoelectron spectroscopy (XPS) of the CsPbBr_3_ film. (c) Energy disperse spectroscopy (EDS) fit curve to show the relative percentage of atoms. Adapted with permission from ref. 21. Copyright 2021 AIP Publishing. (d) Evolution curves of band gap for triple-halide films with increased ratio of Cl/(I + Br + Cl). (e) Evolution curves of host perovskite lattice constant with increasing ratios of Cl/(I + Br + Cl). Adapted with permission from ref. 185. Copyright 2020 The American Association for the Advancement of Science.

In MHPs with the ABX_3_ structure, the metal-halide cage of the BX_6_ octahedron forms a lattice around cation A with relatively weak metal–halide bonds. This soft lattice is susceptible to the structural disturbances associated with point defects.^177^ The high density and diffusion of ion defects can lead to hysteresis of the current–voltage curve, a large dielectric constant, and photophase separation, which can affect the performance of PSCs.^178–181^ As the stoichiometric ratio increases, the grain size decreases (Fig. 16a).^175^ A sub-stoichiometric layer contained larger grains with smaller grain boundary areas, whereas an over-stoichiometric layer contained smaller grains with larger grain boundary areas. The flow ion density and total ion defect density decreased with increasing grain size. This trend suggests that the observed defects were primarily located within the bulk, although it is crucial to note that the grain size variation was achieved through a fractional deviation in the precursor stoichiometry.^182^ Therefore, the relative contributions of the grain volume and grain boundary cannot explain the magnitude of the change in the ion defect density. In contrast, the change in defect density is a direct result of the influence of chemometrics on the defects.^114^ The formation enthalpy exhibited changes corresponding to the grain size, indicating that samples with larger grains and smaller grain boundary areas require more energy to form defects, and *vice versa*. A similar trend was observed for the enthalpy of migration, signifying that the energy barrier for ions to jump between lattice sites decreases with decreasing grain size.^114,183,184^

MHPs commonly contain organic cations such as MA^+^ and FA^+^ and classical inorganic cations such as Cs^+^, resulting in two categories: Cs-based fully inorganic MHPs and organic cation-based MHPs.^18^ For instance, RF sputtering was used to grow large-area CsPbBr_3_ films with uniform structures and compositions (Fig. 16b and c).^21^ Temperature-dependent resistivity measurements revealed typical heat-activated electrical behavior and a band gap of 2.24 eV was obtained, providing insights into the potential large-scale commercial production of PSCs.^21^ Trihalide alloys (chlorine, bromine, and iodine) were used to adjust the bandgap and stabilize the semiconductor under illumination (Fig. 16d and e).^185^ The substitution of iodine with bromine enhances chlorine solubility by reducing the lattice parameter, resulting in the doubling of the photocarrier lifetime and charge-carrier mobility.^161^ The photoinduced phase separation in the film was inhibited even at 1-sun condition, and the degradation rate of the translucent top battery was less than 4% after 1000 h of operation at the MPP and 60 °C.^185^ A PCE of 27% was achieved using a double-ended monolithic series with an area of 1 cm^2^.^185^ Both mixed organic–inorganic and metallic perovskites are variants of the same basic structure.^186^ The perovskite structure can be regulated by changing the elements and structural parameters at the A, B, and X sites to form more variants and derivatives. This inherent flexibility makes perovskites highly interesting and valuable for scientific and engineering applications.

### 5.2. Pressure-induced enhancement

Pressure is a unique variable that can be used to control the electronic structure and performance of organic–inorganic PSCs.^187^ The application of pressure induces dense packing and reduces the atomic distances, potentially leading to alterations in the electron orbitals and bonding patterns. Therefore, the application of pressure can alter the structural, optical, magnetic, and electronic properties of both organic and inorganic solids. Furthermore, an appropriate pressure can strengthen the contact between the layers within the solar cell structure, offering a means of impeding crack propagation along the interfaces.^188^ Consequently, understanding the effect of pressure on the perovskite material layer enables the improvement of material properties through compression. Unlike temperature, pressure did not appear to exert a significant influence on the grain size. These data can be described by the Turnbull model of solid grain growth,^168^ which averages the grain size data collected from films under different pressures at each given processing temperature:5
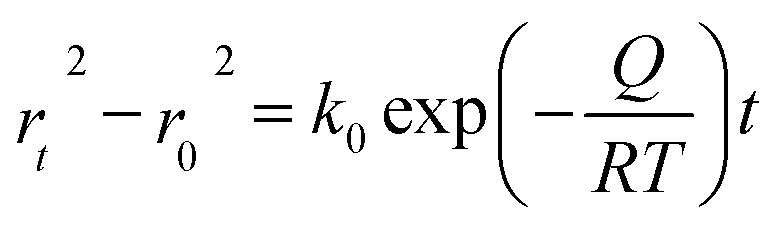
where *r*_0_ is the average grain radius before coarsening (here, the average grain size before hot pressing), *r*_*t*_ is the average grain size at time *t* (60 s), *T* is the process temperature, and *R* is the universal gas constant. The fitting parameters are the pre-reference constant *k*_0_ and molar activation energy *Q* of grain-boundary movement, which is related to the solid-state diffusion of the relevant substance.^189^ Under high-pressure conditions, the photoelectric characteristics remained robust even at elevated temperatures, accompanied by an increase in the series resistance. By controlling the growth conditions and regulating the concentration of shallow defects in the donor or recipient material, the inherent conductivity of MAPbI_3_ can be modified from p-type to n-type.^82^ For example, recent studies have shown that exposure to excess I_2_ vapor pressure can produce intrinsic p-type conduction in MAPbI_3_.^190,191^

**Fig. 17:**
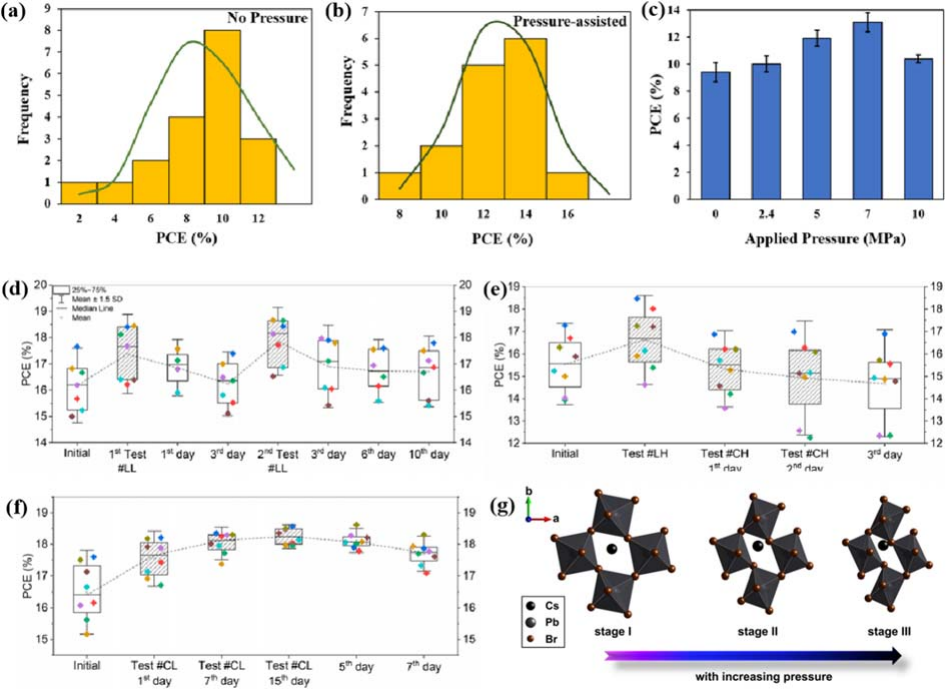
(a–c) Effects of pressure on PCE of PSCs. Adapted with permission from ref. 31. Copyright 2020 Springer Nature. (d–f) PCE test of LL, LH, CH, CL with different time. LL/LH: laminator pressing at low/high pressing pressure, CH/CL: clamp pressing at high/low pressing pressure. Adapted with permission from ref. 30. Copyright 2019 American Chemical Society. (g) Schematic illustrations with respect to polyhedral views of crystal structures of CsPbBr_3_ perovskites under high pressure. Adapted with permission from ref. 187. Copyright 2017 American Chemical Society.

The application of pressure significantly enhanced the PCE of the PSCs compared to those without pressure (Fig. 17a–c).^31^ PSC stacks were also thermally pressed on one side, and it was found that continuous pressing at higher pressures degraded device performance (Fig. 17d–f).^30^ Extrusion-induced increases in compound resistance were observed, which were attributed to reduced charge-carrier capture at interfaces and improved ETL/perovskite and/or perovskite/HTL interfaces, leading to a reduction in the cell hysteresis index after compression.^192^ The primary factor contributing to the enhanced PCE was the increased fill factor, which mainly stems from a decrease in the series resistance. Specifically, 400 mbar pressure was effective for encapsulating PSCs.^193^ In contrast, the excessive pressure of 1000 mbar commonly used for commercial silicon solar modules can lead to the “sinking” of particles at the interface,^187^ potentially damaging the layers and reducing overall system efficiency. Pressure drives structural changes in perovskites, and the redefinition of the boundary conditions of the electron wave function inevitably affects the optical properties.^194^ For instance, in the inorganic trihalide perovskite CsPbBr_3_, the bivalent Pb cation is octahedrally coordinated by the halide Br anion (Fig. 17g).^187^ Below 2 GPa of applied pressure, the bond lengths and angles were shortened under high pressure, resulting in a condensed lead bromide octahedron. After further compression, the compound eventually evolved into an indirect bandgap structure owing to considerable distortion of the more compact octahedral skeleton to accommodate the Jahn–Teller effect. This highlights the close relationship between the band conformation of the perovskite and the crystal structure. This is consistent with the previous finding that excessive pressure can reduce the performance of PSCs.

**Fig. 18:**
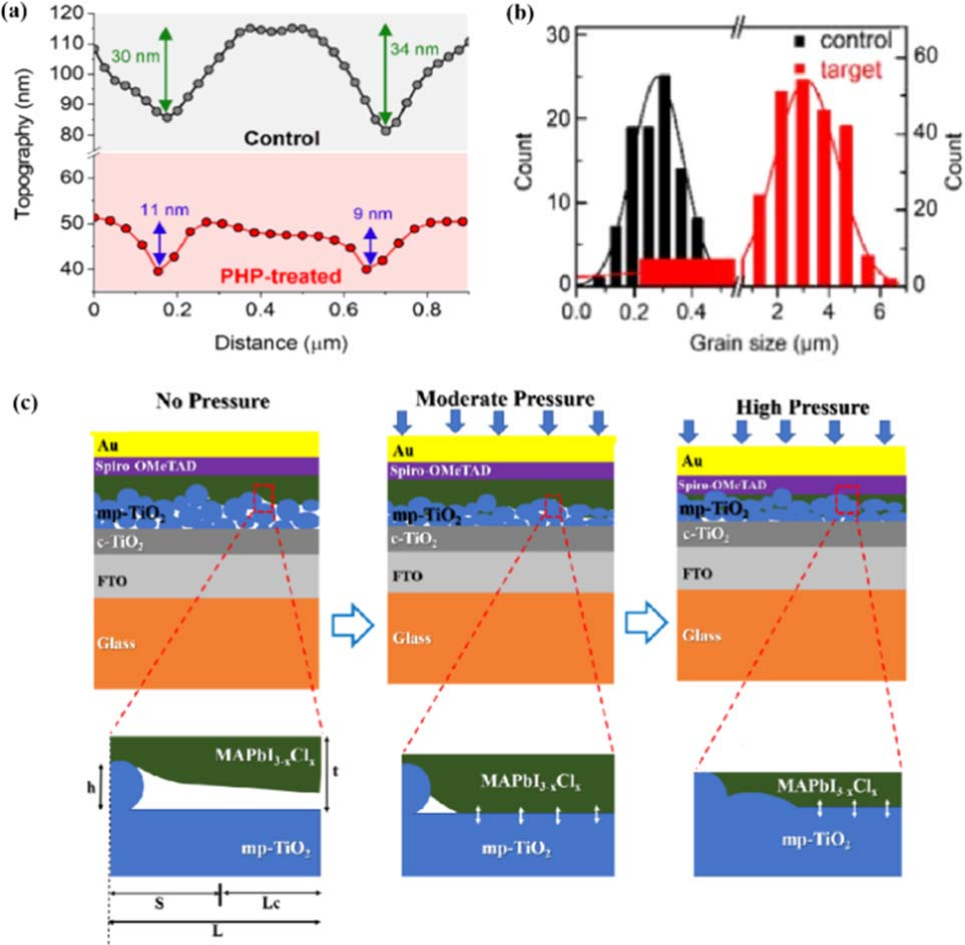
(a) Post heat pressing-treated samples with RMS values. Adapted with permission from ref. 103. Copyright 2022 American Chemical Society. (b) Statistical distribution of domain grain size. Adapted with permission from ref. 33. Copyright 2021 John Wiley and Sons. (c) Schematics of the interfacial surface contact. Adapted with permission from ref. 31. Copyright 2020 Springer Nature.

The grain boundaries of the samples treated with post-heat pressing (PHP) showed lower depths and narrower widths.^111^ Although the exact geometry of grain boundaries measured by atomic force microscopy (AFM) may be influenced by tip surface convolution effects, conducting comparative studies with statistical averages from the same tip mitigates this concern.^195^ The findings of these studies further confirmed that PHP treatment induced the in-plane growth of the film, restructured the grain boundaries from a porous to dense structure, and reduced structural inhomogeneity. Similarly, the average domain size of a target perovskite film after HPS was 3.22 μm, while that of the control perovskite film was only 283 nm.^33^ Larger grains form wider grain boundaries, which provide larger interfacial areas that better separate electrons and holes, thereby increasing the PCE of photogenerated charge collection.^53,186^ In traditional spin-coating methods, perovskite films form defective, porous grain boundaries with original distorted structures.^103^ The PHP process involves selective grain growth in the plane under high pressure, which transforms these boundaries into dense and oriented structures. The application of moderate pressure can significantly increase the PCE of PSCs,^185^ which is attributed to the closure of voids and a corresponding increase in the interfacial surface contact length with increasing pressure (Fig. 18a and b).^31^ Applying moderate pressure to PSCs can improve the interfacial contact between the sandwich particle layers. However, at higher pressures, the absorption of trapped impurities/particles can damage the surrounding layer, thereby reducing the light-conversion efficiency of the solar cell (Fig. 18c). Therefore, selecting the appropriate pressure for hot-pressing PSCs is crucial.

**Fig. 19:**
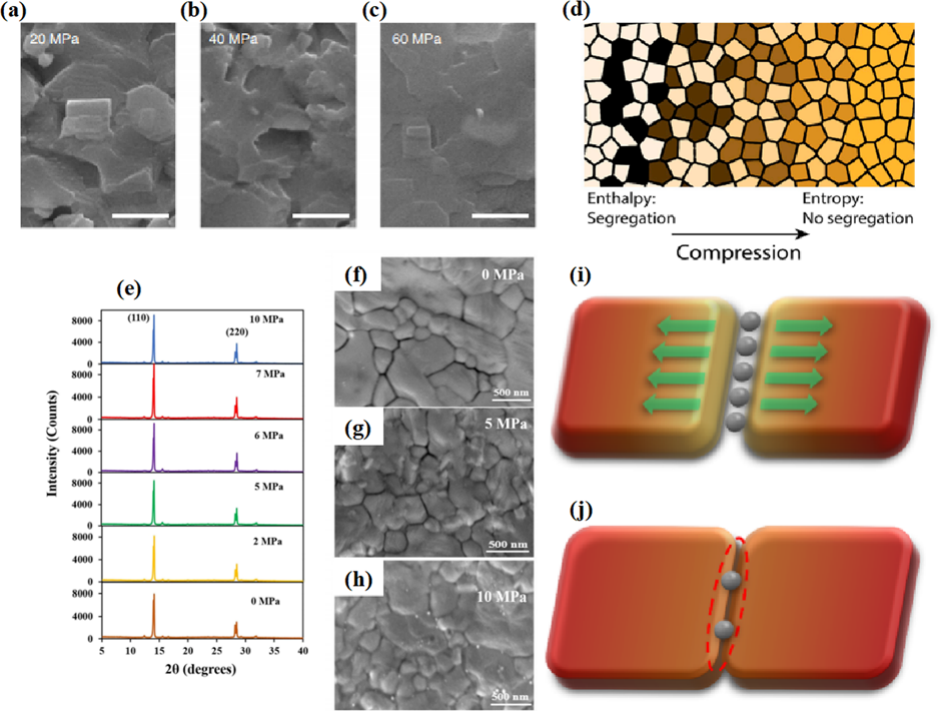
(a) Solid-state in-plane growth-processed 2D/3D films after 10 min at (a) 20 MPa, (b) 40 MPa and (c) 60 MPa. Adapted with permission from ref. 75. Copyright 2021 Springer Nature. (d) Schematic representation of the suppression of segregation by compressing the mixed-halide perovskite. Adapted with permission from ref. 172. Copyright 2020 Elsevier. (e) XRD patterns before and after pressure application. (f–h) Scanning electron microscope (SEM) images of pressure-assisted perovskite films. Adapted with permission from ref. 31. Copyright 2020 Springer Nature. (i) Schematic illustration of phase segregation originated from grain boundaries of the control sample and (j) constrained mobile defects at the grain boundary for the post heat pressing-treated sample. Adapted with permission from ref. 103. Copyright 2022 American Chemical Society.

**Fig. 20:**
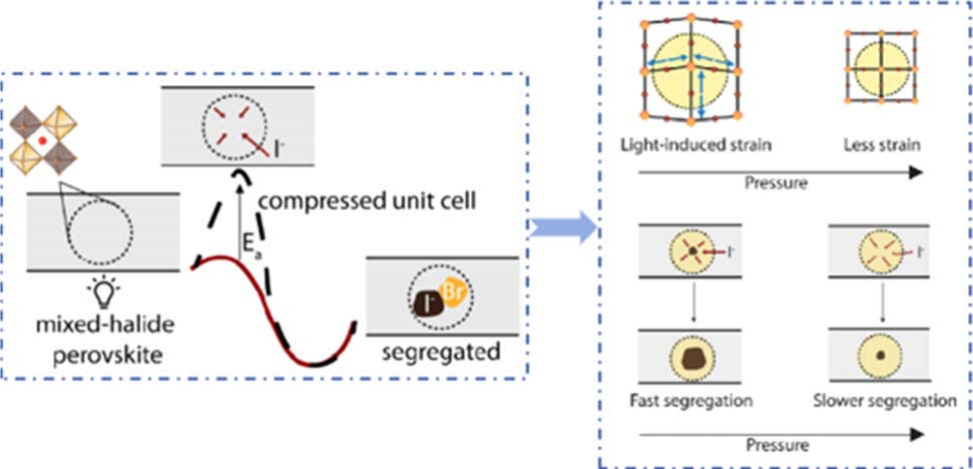
Mechanism scheme of phase segregation stabilization by external physical pressure: high pressure increases the activation energy of halide migration, high-pressure suppression of phase segregation. Adapted with permission from ref. 170. Copyright 2020 American Chemical Society.

It was showed before the surface morphology of a perovskite under different pressures (Fig. 19a–c).^75^ Under 20 MPa, the 2D crystals remained in the process of formation, whereas under 60 MPa, the 2D crystals were nearly flattened, indicating that excessive pressure can reduce the crystallinity of the perovskite surface. In addition, the high compressibility of iodide resulted in a lower ion-size mismatch with decreasing pressure, reducing the microscopic strain in the mixed system (Fig. 19d).^172^ In the range of 0–7 MPa, the (110) and (220) XRD peaks increased with increasing pressure.^31^ The initial pressure increase induced crystallization, leading to bond length shortening and stress-induced phase transitions, which increased the percentage of (110)- and (220)-oriented perovskite crystal phases. However, with a further increase in the pressure (>7 MPa), the (110) and (220) peak intensities decreased (Fig. 19e) owing to the potential stress-induced amorphous phases caused by cracking and damage.^196^ The local amorphous phases reduced the overall crystallinity (Fig. 19f–h). Crystal defects at the grain boundaries and strain lattices allowed active ions to migrate from the grain boundaries, thus accelerating halide segregation (Fig. 19i). The reduction in voids and mobile ions at the grain boundaries can limit the formation of mobile defects at grain boundaries, thus reducing halide segregation (Fig. 19j).^103^ The rate of phase segregation is largely dependent on the pressure applied during PHP, suggesting that strain plays a significant role in ion migration. Under ambient pressures, a similar reduction in phase segregation can be achieved by inducing strain by regulating the strain in the perovskite film through a charge-transport layer or by compositional engineering by partially replacing MA^+^ with a smaller cation.^176^ The strain also plays a key role in the stability of mixed-cationic mixed-halide perovskite compositions, which tend to be less sensitive to photothermal expansion and phase separation.^185,197–199^ Muscarella *et al.* proposed that light causes thermal expansion strain by weakening Pb–X bonds, which reduces the energy barrier of halide migration.^170^ Under high pressure, the compressive strain cancels out the photoinduced strain and increases the activation energy of halide migration. Under ambient pressure, I ions move mainly to the illuminated region (dashed circle; Fig. 20) to increase the volume of the rapidly forming I-rich islands. Under high pressure, owing to the high activation energy, halide ions move slowly, resulting in slower phase segregation.

**Fig. 21:**
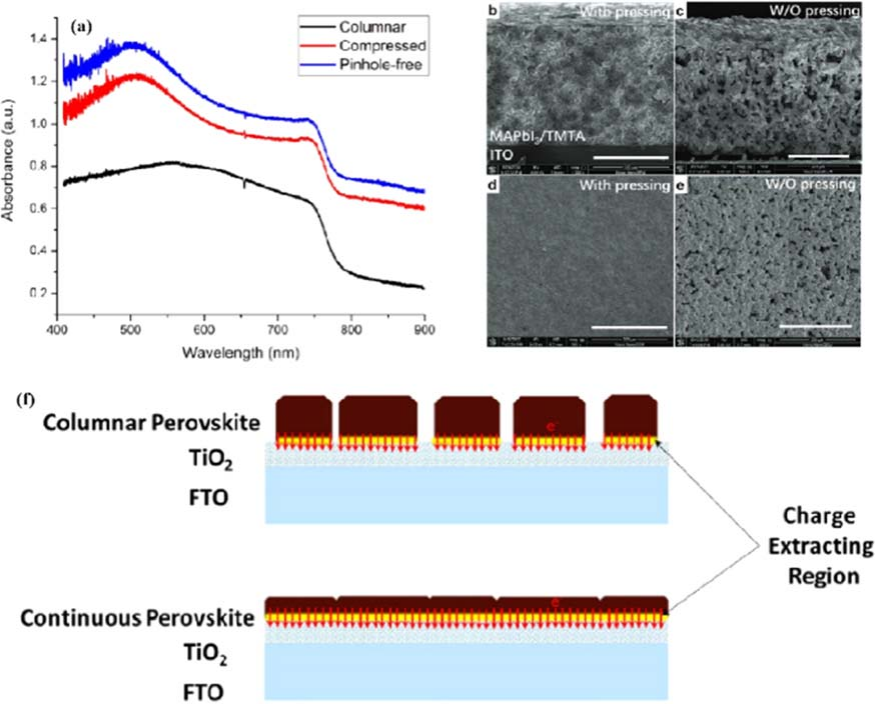
(a) Absorbance spectra of layers. Adapted with permission from ref. 200. Copyright 2016 American Chemical Society. Cross-sectional SEM images of MAPbI_3_/TMTA thick films: (b) with soft-pressing (the scale bar is 100 μm) and (c) without pressing (the scale bar is 300 μm). Surface SEM of MAPbI_3_/TMTA thick films (d) with soft-pressing and (e) without pressing. Adapted with permission from ref. 42. Copyright 2022 John Wiley and Sons. (f) Schematic diagram of active area in charge extraction by columnar and continuous perovskite structure. Adapted with permission from ref. 200. Copyright 2016 American Chemical Society.

Perovskite layers processed under applied pressure exhibited superior absorbance capacity and were characterized as continuous, uniform, and dense structures (Fig. 21a).^200^ This is consistent with the observation that the soft pressing of perovskite films removed pinholes and rearranged the structure to obtain uniform and dense perovskite layers (Fig. 21b–e).^42^ Furthermore, cold-rolling and compression increased the contact area between the perovskite and the TiO_2_ layer.^200^ After treatment, the perovskite layer changed from a columnar microstructure containing a large amount of uncoated TiO_2_ to a continuous and mostly pinhole-free structure and the PCE of the cell increased from 8.16% to 13.24%. This remarkable 62% increase in cell performance was a result of enhanced charge extraction (Fig. 21f). This emphasizes the significance of increasing the contact surface area between the layers.^201^ Therefore, in addition to the various hot-pressing methods discussed earlier, the novel cold-rolling pressing technique presented here is a promising avenue for improving the performance of future PSCs.

### 5.3. Temperature-driven enhancement

Perovskites exhibit rapid degradation at elevated temperatures, which is primarily attributed to structural instability induced by electromigration, ion migration, and interfacial interactions.^202^ Notably, as the temperature increased, iodine began to diffuse significantly below 150 °C, and high temperatures around 175 °C induced lead migration, leading to perovskite degradation and the formation of PbI_2_.^202^ Ion migration leads to poor long-term stability owing to significant structural changes such as lattice distortion and phase decomposition.^203,204^ Additionally, the different distributions of ions in PSCs create different electric field distributions within the semiconductor, resulting in different device performances. Upon heating, two of the three reaction products exhibit reversibility below 100 °C and can release gas in standard geometry, promoting the reaction, consistent with le Chatelier's principle.^205–207^ Moreover, temperature-induced decomposition at approximately 100 °C and phase transition at approximately 57 °C act as catalysts for additional degradation processes.^208^ The lamination process should encapsulate the product formed by thermal decomposition between the two glass substrates, balancing eqn (6) and preventing it from continuing. A direct correlation exists between the hot-pressing temperature and the final grain size. The grain growth is moderated by ion diffusion.^168^ After annealing, all perovskite films had large grain sizes but maintained a uniform, pinhole-free, and flat morphology. However, films treated with HPS displayed larger single-layer particles, indicating that halide segregation was inhibited.

6 CH_3_NH_3_PbI_3_ (s) + heat ⇌ CH_3_NH_2_ (g) + HI (g) + PbI_2_ (s)

**Fig. 22:**
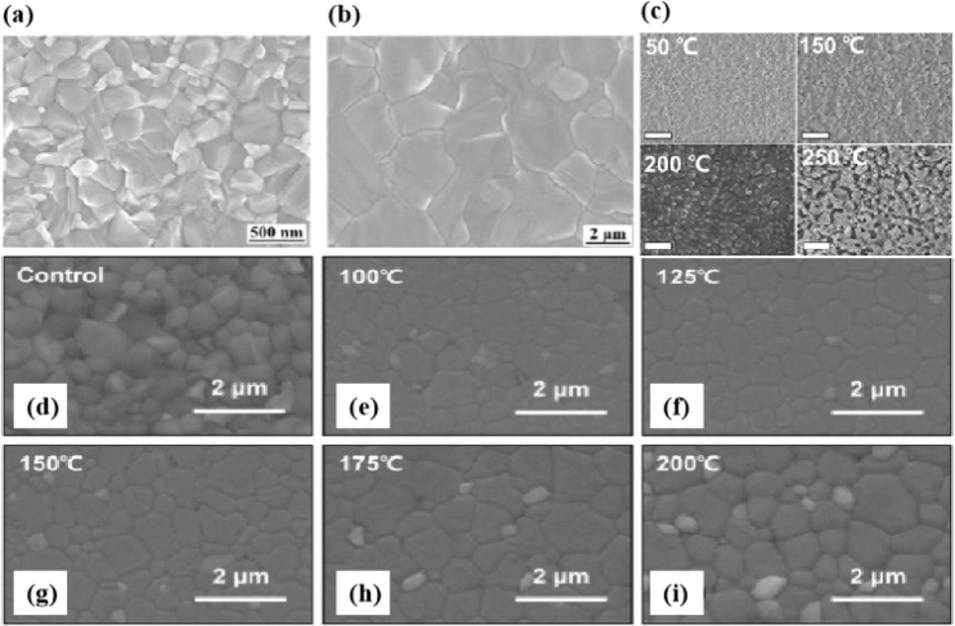
SEM images for (a) the control perovskite film annealed under normal conditions and (b) the target perovskite film annealed under saturated MAI vapor. The perovskite film prepared by the gas flow-induced gas pump method; “target” represents the perovskite film with PbCl_2_ additive treated by the HPS method. Adapted with permission from ref. 33. Copyright 2021 John Wiley and Sons. (c) The SEM images of the films grown under different substrate temperatures, 50–250 °C. Scale bar: 1 μm. Adapted with permission from ref. 21. Copyright 2021 AIP Publishing. (d–i) Field emission scanning electron microscopy (FE-SEM) plane-view images of control and post heat pressing-treated MHP. Adapted with permission from ref. 103. Copyright 2022 American Chemical Society.

In a previous study, a control sample exhibited an average grain size of 501 nm with moderate surface roughness.^103^ Following PHP treatment at 100 °C and 125 °C, the samples exhibited similar average grain sizes to that of the control, yet the surface was flattened under elevated pressure. Notably, at PHP treatment temperatures above 150 °C (the critical temperature for grain growth) (Fig. 22a and b), a significant increase in average grain size was observed.^33^ Although grain growth is typically isotropic, the PHP treatment hinders out-of-plane growth because of the high-pressure environment, which restricts grain expansion in the in-plane direction.^111,209^ Annealing without external pressure does not induce alterations in the internal perovskite structure, and halide segregation can only be caused by internal strain in PHP treatment with sufficient heat treatment at high pressure.^185^ Similar findings were reported for a film comprised of small crystal particles with a columnar structure (Fig. 22c).^21^ During the sputtering of CsPbBr_3_ films, substrate temperatures of 50–200 °C resulted in the complete coverage on the substrate with a pinhole-free film (Fig. 22d–i).^103^ Lower temperatures yielded smaller, disordered crystal particles filling the grain boundaries, whereas increasing the substrate temperature gradually increased the particle size. At growth temperatures above 250 °C, the film surface became rough with prominent grain boundaries and holes.^210^ Therefore, the substrate temperature has a clear effect on the quality of RF magnetron-sputtered films. Rapid crystal growth and insufficient particle diffusion at high temperatures were the main reasons for the growth of uneven films. According to classical nucleation theory, the nucleation and growth of crystals can be explained by differences in the Gibbs free energy due to changes in the volume and surface area of newly formed crystals.^211^

**Fig. 23:**
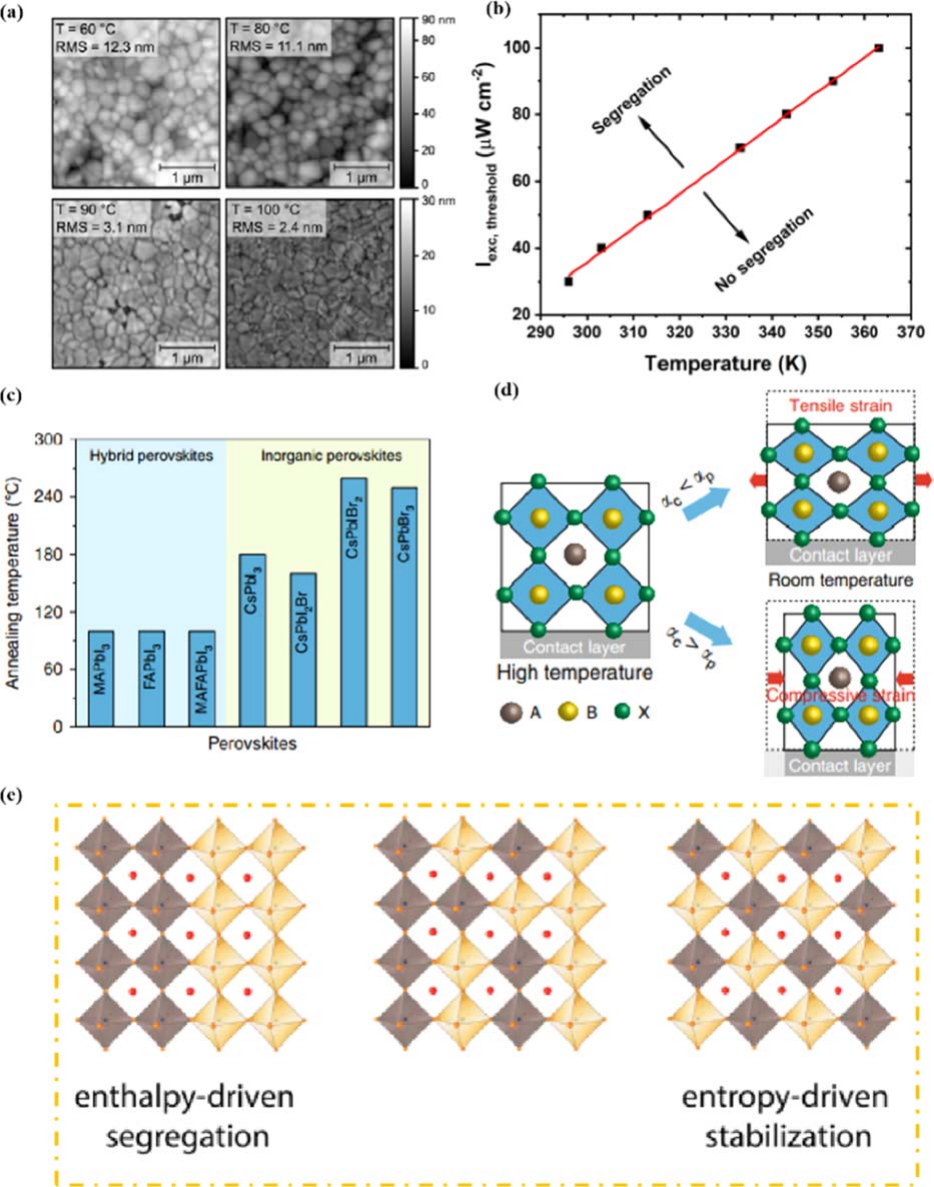
(a) AFM images of delaminated perovskite surfaces. Adapted with permission from ref. 169. Copyright 2020 John Wiley and Sons. (b) *I*_exc, threshold_ as a function of temperature for observing halide ion segregation in MAPbBr_1.5_I_1.5_ mixed halide perovskite films. Adapted with permission from ref. 112. Copyright 2020 American Chemical Society. (c) Annealing temperatures of different hybrid and inorganic perovskite films during formation. (d) Schematic showing the formation of tensile and compressive strains. Adapted with permission from ref. 176. Copyright 2020 Springer Nature. (e) Diagram of enthalpy driven separation process and entropy driven remixing process. Adapted with permission from ref. 172. Copyright 2020 Elsevier.

To analyze perovskite recrystallization during lamination, AFM images of delaminated PSCs previously processed through lamination were analyzed.^169^ The layer roughness decreased with increasing lamination temperature. Optimal device performance occurred at 90 °C, aligning with the temperature employed during nanoimprinting of the perovskite layers.^211–214^ At this temperature, the perovskite recrystallized into an exceptionally smooth layer with a root-mean-square roughness of ∼3.1 nm (Fig. 23a).^169^ A subsequent increase in the lamination temperature further reduced the RMS roughness but did not yield a higher PCE. The crystal structure of the tricationic perovskite remained unchanged under the combined effects of high pressure and temperature. The mixing (or homogenization) process accelerates with increasing temperature, indicating that a thermally activated mechanism governs the mixing/exchange of halide ions. Elmelund *et al.* summarized the observed temperature-dependent excitation intensity thresholds and indicated that the excitation intensity values for inducing (inhibiting) photoinduced halide segregation at a given temperature were higher (lower) (Fig. 23b).^112^ The existence of an excitation intensity threshold for phase segregation refutes the polaron strain model.^131^ Mixing entropy explains the thermally activated mixing of halide ions to produce mixed-halide perovskites.^124,215^ When the mixed halide perovskite phase is separated, the I-rich domains act as the recombination sites for photogenerated carriers, which limits the performance of halide PSCs.^216,217^

The perovskite films in high-efficiency PSCs typically need to be annealed at temperatures above 100 °C to improve their crystallinity and minimize defects.^218–222^ Notably, all-inorganic perovskites require higher temperatures than hybrid organic–inorganic perovskites to stabilize the black cubic perovskite phase (Fig. 23c). In particular, CsPbI_3_ requires an annealing temperature of 180 to 330 °C.^218,223^ Inorganic perovskite films processed at high temperatures are subjected to greater tensile strain. When the perovskite film is applied to a layer with a low *α* (coefficient of thermal expansion), the contact established between the layers during high-temperature annealing restricts perovskite shrinkage upon cooling to room temperature, thereby introducing tensile strain along the in-plane direction (Fig. 23d). Conversely, the use of layers with high *α* values results in higher shrinkage of the perovskite film, resulting in compressive strain. Thus, the regulation of tensile or compressive strain in perovskite films can be achieved by selecting adjacent device layers with lower or higher *α* compared to perovskite.^176^ Light-induced halide segregation is reversible owing to thermal activation, allowing the separated region to return to its original uniform composition and the film to fully recover in dark conditions.^112^Fig. 23e shows enthalpy driving the separation process, whereas the entropy drives the remixing process.^172^ Defects arising from halide-ion vacancies seem to contribute to the separation of halide ions.^2^ Hole trapping by iodide ions also induces halide ion migration, requiring that the thermodynamic activation barrier is overcome to prevent the halide ions from recombining.^112^ Formula (7) describes the migration of halide ions in the opposite direction.^112^

7 (*n*/2)MAPbBr_3_ + (*n*/2)MAPbI_3_ → *n*MAPbBr_1.5_I_1.5_

This illustrates the thermal-activation-induced exchange of Br and I in mixed films of MAPbBr_3_ and MAPbI_3_.^123^ Similarly, a pre-separated mixed halide ion membrane required 14 h to fully recover at room temperature, indicating the slow migration of halide ions.^112^ Nevertheless, as the temperature increases, the rate of dark reduction (mixing of halide ions in the original mixed halide composition) significantly increases.

**Fig. 24:**
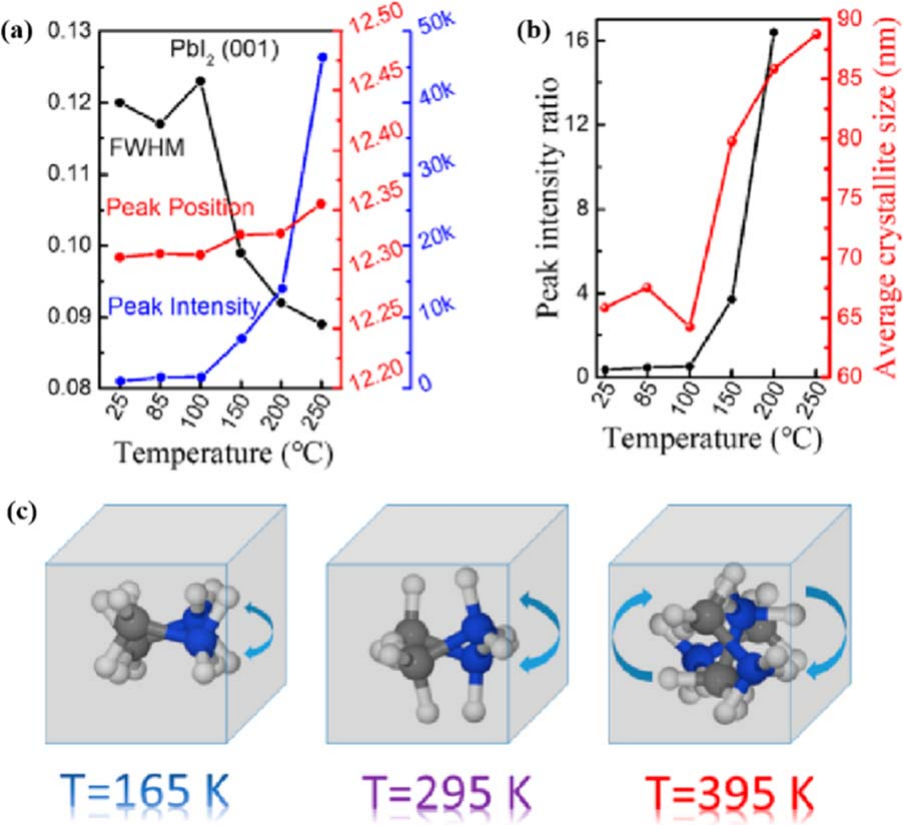
(a) FWHM, peak positions, and intensities of the curves. (b) Peak intensity ratio and average crystallite size of the curves. Adapted with permission from ref. 107. Copyright 2020 Springer Nature. (c) Scheme of the rotational state for the MA^+^ at different temperatures. Adapted with permission from ref. 119. Copyright 2018 American Chemical Society.

For a given plane, a narrower crystal plane spacing implies a more tightly packed atomic arrangement. In addition, the elevated intensity of the PbI_2_ (001) peak with increasing temperature (Fig. 24a) indicates an increase in the crystallinity of PbI_2_.^107^ According to Scherrer's equation,^107^ the full width at half maximum (FWHM) of the peak is inversely proportional to the crystal size. A decrease in the FWHM indicated an increase in the grain size of PbI_2_ from 65.84 nm to 88.78 nm.^107^ Furthermore, the ratio of the PbI_2_ (001) and perovskite (110) peak intensities increased significantly with increasing temperature (Fig. 24b).^107^ This was attributed to the decomposition of the perovskite into PbI_2_ at high treatment temperatures, leading to a significant decline in the performance of PSCs treated at excessively high temperatures. Annealing at temperatures at or above the quadrilateral-to-cubic transition range led to a gradual loss of spontaneous MA^+^ order (Fig. 24c), resulting in a notable increase in the susceptibility to degradation.^224–226^ Addressing this vulnerability in material degradation behavior implies that the durability can be extended by operating the solar device solely in the tetragonal CH_3_NH_3_PbI_3_ region (*i.e.*, just below ∼50 °C) or by increasing the phase-transition temperature through feasible lattice engineering.^119^ A correlation between the intrinsic modification of the perovskite lattice under heating and the interdiffusion of atoms from the boundary material above this thermal threshold is also plausible. It is essential to note that an appropriately timed heat treatment can effectively passivate defects, thereby improving the average PCE of PSCs.

**Fig. 25:**
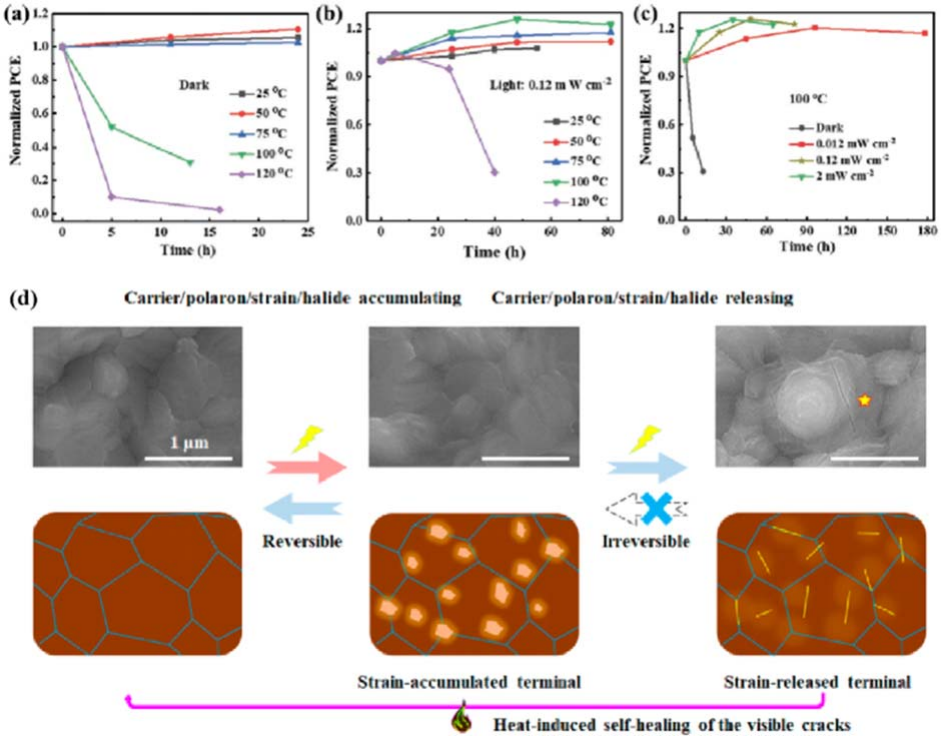
Influence of thermal-annealing temperature on the device performance of quasi-2D PSCs (a) in the dark and (b) light conditions. (c) Influence of light intensity on the performance of quasi-2D PSCs. Adapted with permission from ref. 227. Copyright 2018 American Chemical Society. (d) SEM images and corresponding schematic diagram of strain accumulating/releasing induced photoinduced halide segregation/photoinduced self-healing procedures. Adapted with permission from ref. 111. Copyright 2021 American Chemical Society.

Heat is produced in the PSC under illumination. To elucidate the distinct roles and effects of heat and light in thermal-optical co-processing, changes in device performance under thermal annealing and illumination conditions were investigated (Fig. 25a–c).^227^ These findings confirm the synergistic effect of light and heat treatments in improving device performance. The continuous production of photoexcited carriers leads to lattice distortion attributed to polarons/strain.^228,229^ The strain gradient produced by the resultant driving force accelerates carrier accumulation and remains entirely reversible until the strain-tolerance threshold of the perovskite lattice is reached. However, once the accumulated strain exceeded the strain-tolerance threshold, lattice damage occurred, resulting in the formation of nanoscale cracks throughout the film to release excessive strain (Fig. 25d).^111^ As the strain accumulation process is non-uniform across the polycrystalline perovskite film, it takes time to achieve the complete release of the internal strain. Therefore, the damaged perovskite film after strain relaxation did not exhibit photohalide segregation, offering a plausible explanation for the non-repeatability of polycrystalline MHP films. In summary, it has been shown that cracks self-heal through heat induction.

### 5.4. Doping-related enhancement

Defects present in organic–inorganic halide perovskite films can be addressed through strategies such as passivation using ionic and coordination bonds, use of wide-band-gap materials, and preventing ion migration at extended defects, as depicted in Fig. 11. Semiconductors based on organic–inorganic MHPs adhere to the common ABX_3_ perovskite structure, where A can represent either a cation or an inorganic cation, depending on the ion size and Goldschmidt tolerance factor 
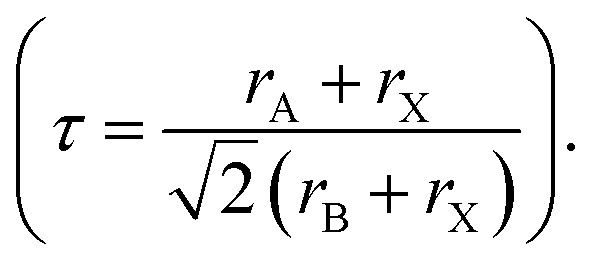
^2,4,90^

Promising outcomes have been observed with the use of organic cations like MA and formamidine (FA).^1^ Adding suitable molecules to perovskites can achieve PSCs with high PCE and thermal stability. For example, the FAPbI_3_ perovskite film degraded quickly after 18 d of exposure to a humid environment, whereas the FA_0.85_Cs_0.15_PbI_3_ film remained stable after the addition of Cs.^230^ Accordingly, the performance of the FA_0.85_Cs_0.15_PbI_3_ device was more stable than that of pure FAPbI_3_ cells. Introducing small amounts of Rb^+^ and K^+^ (*x* ≤ 0.05) to FAPbI_3_ can enhance the performance of the PSCs, but higher concentrations may induce phase separation.^158^ However, PSCs fabricated using a hot-pressing method with a laminated interface for the perovskite film exhibited suboptimal performance, yielding a PCE of 14.4%.^76,169^ To obtain large-grain perovskite films, many researchers have added chloride to the perovskite precursor solution as an effective method for promoting crystallization and grain growth.^231,232^ For example, MACl containing dimethyl sulfoxide was added to obtain a vertical-oriented perovskite framework with low defect density, thus enhancing the PCE of the resulting PSCs.^233^ This additive also enhanced grain-boundary movement, facilitating the formation of a dense grain morphology.^4^ A planar heterojunction PSC with a PCE of 17.10% was achieved by doping PbCl_2_ with MAPbI_3_, demonstrating a promising method for the large-scale production of high-performance PSCs through magnetron sputtering.^24^

Excess PbI_2_ in the perovskite film creates traps that are not conducive to device performance and stability.^24^ Unlike cations, mixing different halides at the X-position can significantly influence the optical and electronic properties, absorption and emission spectra (bandgap), carrier lifetime, and diffusion length.^2^ Chlorine doping changes the surface morphology and grain size of perovskite films by reacting with MAI, which is an effective method for controlling the crystallization of the perovskite phase. A hot-pressing technique induced sublimation of the MAI film, preventing leakage of the MAI vapor.^33^ Introducing an appropriate amount of PbCl_2_ into the perovskite material improves the film quality by slowing the crystallization rate, resulting in a more uniform, pinhole-free film, thereby enhancing device performance and stability. However, excess PbCl_2_ cannot be fully converted into perovskite by MAI post-processing, resulting in pinholes in the final perovskite film.^24^ Chlorformamidine (FACl) was used as an additive to expand the grain size of perovskite films and improve their quality.^232^ The environmental stability of the perovskite film was significantly improved; the device maintained 90% of its initial PCE after 1200 h in ambient air, whereas the original device without FACl treatment maintained only 50% of its initial PCE.^232^ This approach presents a promising pathway for achieving high-performance and stable PSCs through effective crystallization management. The addition of Cs and FA to perovskite materials can improve the phase stability of CsPbI_3_ and inhibit the yellow phase of FAPbI_3_, providing a promising way to improve the PCE and thermal stability. A mixture of large FA and small Cs is conducive to forming the preferred black perovskite phase, removing volatile substances, and improving thermal stability.^234^ By adding MACl to perovskite materials, a PSC including chloride ions was developed, which achieved a PCE of 21.65% after 500 h of illumination, a PCE of 96% at 65 °C, and an initial PCE of 85%.^235^ MACl acts as a crystallization aid to promote grain growth, reduce defects, and form a high-quality perovskite film with excellent photothermal stability. MABr was incorporated into MAPbI_3_ to form a high-quality MAPbI_3−*x*_Br_*x*_ absorber, and the PCE of the device increased from 14–16% to 19.2%.^161^

**Fig. 26:**
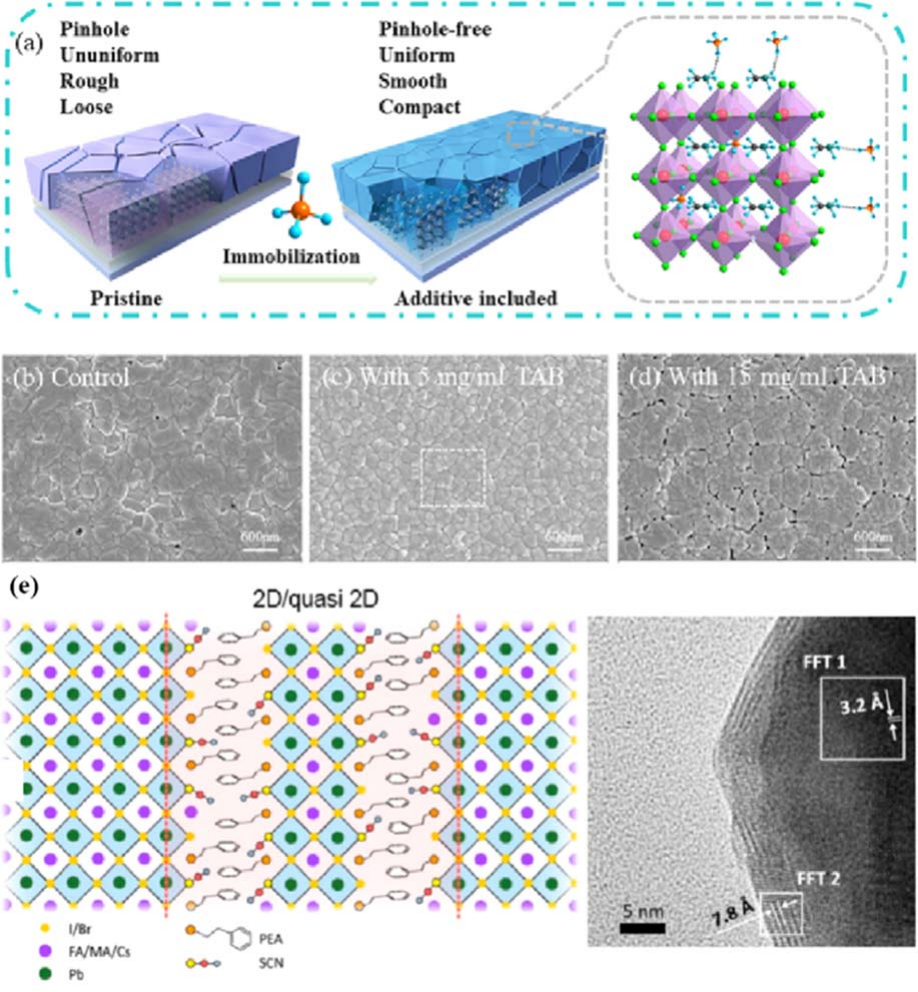
(a) Schematic demonstration of the process of superhalogen BH^4−^-induced morphological changes of the perovskite film. (b–d) Top-view SEM images of perovskite films with 0, 5, and 15 mg mL^−1^ tetrabutylammonium borohydride (TAB) additives deposited on mesoporous-TiO_2_/compact TiO_2_/FTO/glass substrates. Adapted with permission from ref. 78. Copyright 2020 American Chemical Society. (e) Formation mechanism and TEM images of the (quasi-)2D/3D structure constructed using PEAI and Pb(SCN)_2_ as additives. Adapted with permission from ref. 238. Copyright 2019 Elsevier.

The simultaneous integration of cations and anions leads to notable enhancements in both cell efficiency and stability. For instance, the addition of quaternary ammonium halides to perovskite precursor solutions can simultaneously passivate cation (MA) and anion (iodine) vacancies, with quaternary ammonium compensating for cation vacancies and the halide compensating for anion vacancies.^236^ BH^4−^ anions, with a similar radius to that of iodide anions, enable the formation of a stable mixed-halide perovskite structure, MAPbI_3−*x*_(BH^4−^)_*x*_, by partially substituting iodide or compensating iodide vacancies. Notably, BH^4−^, composed of two elements, exhibits chemical properties akin to halogens, earning it the designation of a superhalogen.^78^Fig. 26a shows the effects of additives on the morphology of perovskite films. Iodine is partially replaced by BH^4−^, and all major elements (C, I, N, Pb, and B) are evenly distributed throughout the membrane without obvious phase separation.^78^Fig. 26b–d show top-view SEM images of the MAPbI_3_ perovskite film when 0, 5, and 15 mg mL^−1^ of additives were added.^78^ For 5 mg mL^−1^ additive, a smooth, dense, and pinhole-free film was obtained, whereas concentrations exceeding 10 mg mL^−1^ resulted in island-like formations, indicating additive accumulation at the grain boundaries. Br doping and the mixing of FA and Cs improved the crystallinity of the crystal, increased the grain size, reduced the trap density and degree of recombination, accelerated the extraction and transfer of charge, and ultimately improved the PCE.^162^ By selecting or mixing the optimal concentrations of the appropriate ions at the A and X sites, both high PCE and high light stability can be achieved. Strategic modulation of the FA/Cs and Br/I ratios yielded an FA_0.85_Cs_0.15_PbBr_0.15_I_2.85_ PSC device that maintained a PCE of 19.6% after 500 h of light exposure.^237^ Furthermore, a PEAI additive was introduced into a PSC along with Pb(SCN)_2_, which reduced the defect density and energy disorder, improved the carrier mobility and lifetime, and achieved a PCE of up to 19.8% (Fig. 26e).^238^ This compositional engineering strategy promotes perovskite crystallization, resulting in improved crystal orientation, larger grain sizes, reduced trap densities, and enhanced morphological uniformity.

### 5.5. Passivation-enhanced enhancement

Small organic molecules are widely used materials capable of effectively passivating interfaces in perovskite films by substantially reducing the number of defects, thereby contributing to the development of efficient and stable PSCs. For instance, Noel *et al.* demonstrated for the first time that thiophene can interact with Pb^2+^ atoms on the surface of a perovskite, thus passivating defects on the perovskite surface.^239^ The organic small-molecule pyridyl-2-carboxylead material (PbPyA_2_) improved the light stability of PSC devices, with an initial PCE of 19.96% and a retention rate of 93% after 500 h of MPP tracking.^240^ PbPyA_2_ small molecules promote crystallinity, passivate grain boundaries, improve film quality, and prevent component volatilization. Metal ions and organic cations effectively passivate films through ionic bonds and other electrostatic interactions with the negatively charged defects in perovskite materials.^241^ K^+^ passivates the undercoordinated halides at the surface and grain boundaries through ionic interactions, specifically with undercoordinated Br^−^ and I^−^, fixing excess halides by forming benign compounds at grain boundaries and surfaces. In addition, K^+^ binding with the halides effectively inhibited ion migration. For example, the addition of potassium iodide to perovskite precursors results in K^+^ accumulating on the surface and grain boundaries, passivating extended defects, and yielding an internal photoluminescent quantum efficiency exceeding 95% in perovskite films.^242^

Double-sided passivation layers can greatly increase the *V*_oc_ and provide significant stability,^83^ that is, the PCBM–PMMA layer on the SnO_2_@TiO_2_ layer and the PMMA layer on the perovskite film. This passivation approach involves the use of a polymer material as both a passivating agent and an insulating component for the perovskite, reinforcing the perovskite film surface and shielding it from detrimental external degradation factors. Polymer materials are effective surface enhancers for perovskite films, preventing external degradation. Ligands play an important role in passivating surface defects and/or forming robust bonds with perovskites, thereby protecting the perovskite lattices from degradation. Quantum dots include various elements and ligands and can facilitate surface passivation and interactions with perovskites.^108^ However, the practical applications of organic molecular layers are constrained by the presence of inorganic ions and their inherent chemical instabilities. Stable LDPs have proven to be effective in enhancing the interfacial contact between the light-absorption layer and the HTL.^47,146,243^ Compared to 3D perovskites, LDPs exhibit a higher formation energy, necessitating more energy for removal. This inhibits the degradation of the perovskite absorbers by protecting them against external factors, thereby improving cell stability.^244^

Furthermore, incorporation of 2D materials can reduce the defect density and ion migration, thereby improving device stability. Triethyl tetramine vapor was used to surface-passivate PSCs, which significantly improved their PCE (from 17.07% to 18.03%) and stability (PCE retention from 73.4% to 88.9%).^79^ Furthermore, a novel heterocyclic ammonium salt cation (2-methylthio-2-imidazoline) was used as a low-dimensional perovskite passivation layer to stabilize the perovskite layer and enhance the photovoltaic performance of PSCs.^87^ The amphoteric properties and robust molecular interactions of the MT-Im LDP layers effectively passivated the amphoteric Pb-based defects at the interface, substantially inhibiting the generation of Pb^0^. This resulted in a reduction in interfacial nonradiative recombination, an increase in carrier lifetime, and an overall improvement in device stability. These improvements were attributed to the excellent hydrophobicity of MT-Im and its effective targeted treatment of Pb-based defects at the interfaces.

**Fig. 27:**
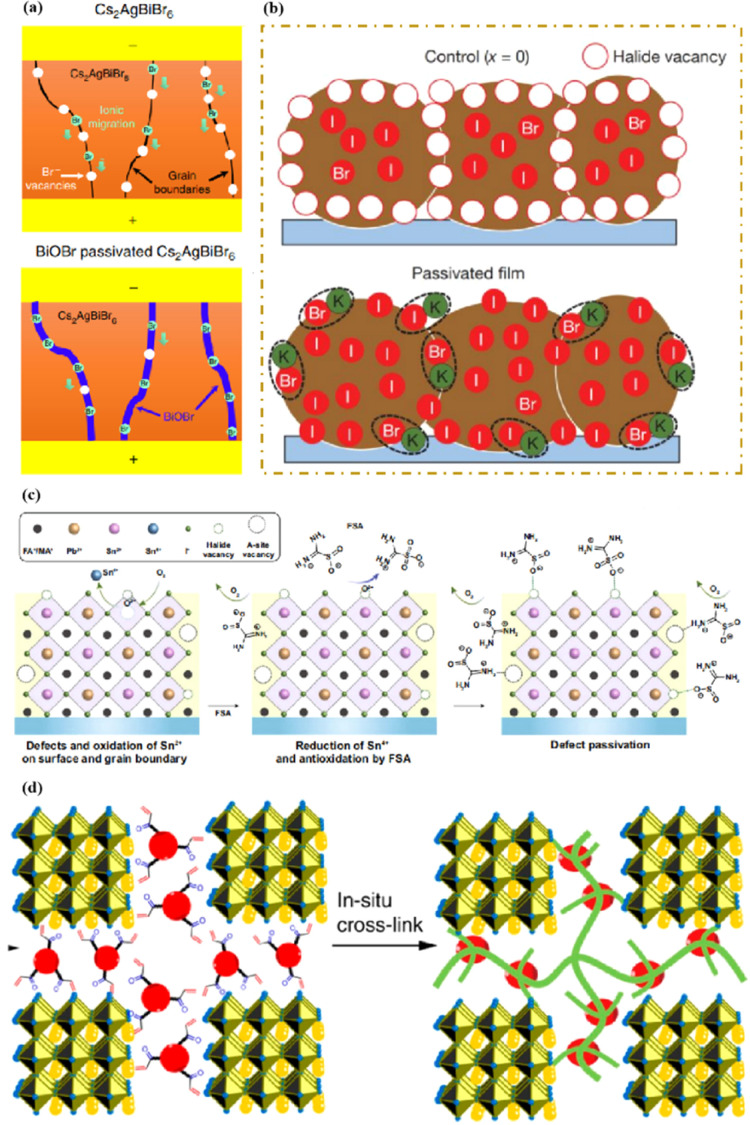
(a) Schematic diagram of BiOBr passivation inhibiting ion migration. Adapted with permission from ref. 59. Copyright 2019 Springer Nature. (b) Schematic cross-section of a film showing halide vacancy management in the case of excess halide. Adapted with permission from ref. 242. Copyright 2018 Springer Nature. (c) Schematic illustration of suppressing Sn^2+^ oxidation and the passivation of halide and cation vacancy at the grain surface of mixed Sn–Pb perovskite films enabled by FSA molecule. A-Site represents the organic monovalent cations in the lattice. Adapted with permission from ref. 251. Copyright 2021 Springer Nature. (d) Cross-linking mechanism using TMTA molecules. Adapted with permission from ref. 136. Copyright 2018 Springer Nature.

Currently, the further development of PSCs is limited by their high grain-boundary density, interfacial defects, and interfacial energy barriers.^245^ The introduction of polymers also facilitates defect passivation and performance enhancement. Among the commonly used additives for passivating grain boundaries or surface defects, alkyl ammonium halides stand out because of their ability to passivate both cationic and anionic defects and enhance passivation through hydrogen/ionic bonds. Son *et al.* first reported the precipitation of excess MAI in grain boundaries, where the MAI layer inhibited nonradiative recombination and improved hole and electron extraction in the grain boundaries by forming effective ion-conduction pathways.^246^ Furthermore, BiOBr was grown epitaxially on Cs_2_AgBiBr_6_ crystals to inhibit surface defects at the grain boundaries of Cs_2_AgBiBr_6_.^247^ In Cs_2_AgBiBr_6_, Br vacancies are the main reason for migration (Fig. 27a and b) and the presence of BiOBr provides Br^−^ to fill these vacancies, thus reducing the number of ion migration channels and ion conductivity. In another study, P123 polymer (PEG–PPG–PEG) was added to a perovskite solution and used to functionalize grain boundaries to form a nanoscale grain-boundary wall, which improved the crystallinity of the perovskite and smoothness of the film.^248^ Despite their high photostability, these polymers are inherently insulating and hinder the extraction and transfer of charge, thereby limiting potential efficiency gains.

To further reduce the defect density in mixed Sn–Pb perovskites, FSA as a potent reducing molecule can be used to effectively reduce Sn^4+^ defects and passivate surface ion vacancies.^249^ Sulfhydryl groups react with oxygen molecules to inhibit the oxidation of Sn^2+^, while the O atoms from the sulfhydryl groups act as electron donors and form coordination bonds with the undercoordinated Sn^2+^ or Pb^2+^ cations, thereby passivating the surface halide vacancies.^250^ Furthermore, the formamidine groups in FSA, with a cationic structure similar to that of FA, passivate the surface A-site vacancy defects (Fig. 27c).^251^ This synergistic effect of surface-anchored FSA increased the carrier recombination lifetime in the Sn–Pb perovskite by *a* factor of three.^251^ A simple and effective general anionic modification strategy for improving the photovoltaic performance of PSCs was proposed, which involves modifying the perovskite/SnO_2_ interface using a series of guanidine salts containing different anions.^245^ All anions played an active role in passivating SnO_2_ and perovskite surface defects, optimizing the interfacial energy band arrangement, and promoting the crystallization of PbI_2_ and perovskite. Different modifiers exhibited varying degrees of positive effects, which can be fine-tuned by selecting the anion types and interfacial chemical interactions. Interface modification improves the thermal and environmental stability of the perovskite. Cross-linked particles incorporating functional organic molecules have proven to be effective in passivating defects/imperfections on the grain boundaries of perovskite layers. Trimethylpropane triacrylate (TMTA) was incorporated into the MAPbI_3_ layer, where the TMTA molecules were chemically anchored to the grain boundaries and crosslinked *in situ* to form a robust continuous polymer network after thermal annealing (Fig. 27d).^136^ The addition of TMTA improved the PCE by passivating defects. Notably, the cross-links formed by the TMTA molecules significantly enhanced the stability of the device against ultraviolet light, temperature, and moisture. The increased PCE was primarily attributed to an increase in the open-circuit voltage.

**Fig. 28:**
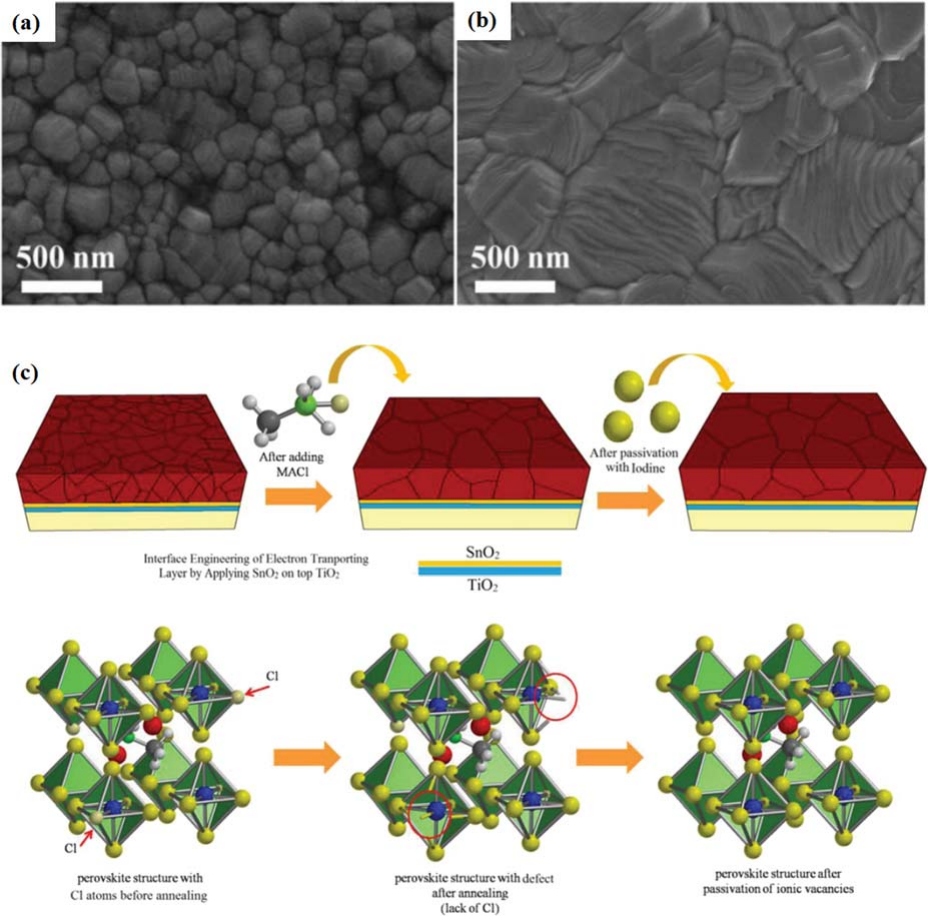
Top-view SEM images of triple A-cation perovskite films (a) without and (b) with MACl additive in the starting solution. (c) Schematics of the triple A-cation perovskite films without MACl, with MACl additive and after passivation with iodine. Adapted with permission from ref. 235. Copyright 2018 John Wiley and Sons.

Techniques for enhancing the PCE of PSCs include the incorporation of additives that simultaneously dope and passivate the materials. For example, the effect of adding MACl to Cs/MA/FA precursor solutions was shown to reduce the nucleation rate and produce strong interconnected films with larger grains.^235^ The incorporation of MACl led to a remarkable increase in the grain size from 200 nm to over 1000 nm (Fig. 28a and b).^235^ Chloride ions, which are mobile within halide perovskite films, play a crucial role in crystal formation and growth. The Cl atoms leave the perovskite lattice in the form of MACl, resulting in ion vacancies, particularly on the perovskite surface.^236^ The surface traps were further reduced by passivation with an isopropyl alcohol iodine solution diluted with chlorobenzene (Fig. 28c).^235^ The passivation process resulted in an extended carrier lifetime, contributing to a high *V*_oc_ of approximately 1.24 V, with a minimal potential loss of approximately 370 mV.^235^ Furthermore, the PSCs exhibited an impressive PCE of 21.65% and robust stability.^235^ Notably, the distribution of chloride ions within the perovskite layer was non-uniform and predominantly located deep within the layer. It was reported that chlorine was released from the surface of the solar cell after the formation of the perovskite film.^135^

The efficient transfer of photo-excited electrons and holes is crucial for achieving effective solar energy conversion. A simple and effective acid treatment strategy was used to weaken the zigzag octahedral chain bonds in anatase TiO_2_ and shorten the crisscrossing octahedral chains, thereby creating an amorphous TiO_2_ buffer layer that was tightly bound to the anatase surface and acted as an ETL for efficient electron transport.^252^ The amorphous TiO_2_ ETL had a higher electron density owing to the presence of oxygen vacancies, resulting in the efficient transfer of electrons from the perovskite to TiO_2_. Compared to the as-prepared TiO_2_-based devices, the acid-treated PSCs exhibited a higher short-circuit current and PCE. Furthermore, an exceptionally high PCE was achieved for laminated PSCs with the surface and interfacial grain boundaries of the perovskite films treated with acetonitrile (ACN; a polar aprotic solvent).^76^ After ACN treatment, the perovskite film surface became smooth, the grain boundaries dissolved, and the grain boundary interfaces widened. The smooth surface minimizes the probability of local contact between the top and bottom perovskite films, effectively suppressing hole defects. The hot-pressing process enabled uniform elastoplastic deformation of the perovskite films, resulting in an outstanding PCE of 22.52%. The PSCs exhibited long-term stability, maintaining an average of 96% of their initial PCE for over 2000 h. The composition of the perovskite films did not change under the applied pressure and heat. In another study, 2,5-diphenyl C_60_ fullerpyrrolidine (DPC_60_) was used as an interface bridge for highly efficient planar PSCs with enhanced stability.^132^ The hydrophobic DPC_60_ layer inhibited the heterogeneous core of the perovskite films, thereby improving the crystallinity and stability of the device. Compared to PSCs without the hydrophobic DPC_60_ layer, the efficiency was 8% higher. After 200 h of continuous solar irradiation and thermal aging at 55 ± 5 °C, the devices maintained 82% of their initial PCE.

Introducing quantum dots is another promising approach for developing PSCs with high PCE and thermal stability. Fluorographene quantum dots (FGQDs) have been used to passivate defects in perovskite films *via* the strong interaction between fluorine atoms from the FGQGs (with exposed ions and groups) and the perovskite, thereby reducing the trap density, inhibiting non-radiative recombination, and enhancing charge mobility.^48^ Adding a protective insulation layer to PSC devices is also an effective strategy for enhancing stability as it minimizes the ingress of external degradation factors such as moisture and oxygen. One of the main causes of light and thermal instabilities is ion migration, which alters the chemical composition of perovskites. An effective protective layer plays an important role in inhibiting ion migration and chemical reactions.^108^ In addition, good protective layers exhibit potent passivation effects, reduce defects, suppress recombination, and facilitate the formation of uniform, highly crystalline, and high-quality films. Such protective layers have emerged as crucial tools for performance improvement *via* interfacial engineering, including composition engineering, which is particularly suitable for delicate perovskite materials and other organic/hybrid materials.^108^ Therefore, to obtain a stable high-performance PCE, it is necessary to both passivate the interfacial defects that inhibit non-radiative recombination and minimize long-term stable ion migration in different environments.^87^ In summary, a reduction in the unit-cell volume achieved through physical pressure, heating, or component engineering can effectively delay the migration of halides by increasing the activation barrier of the migration process, thereby improving the performance of PSCs.

## 6. Structure simulation

**Fig. 29:**
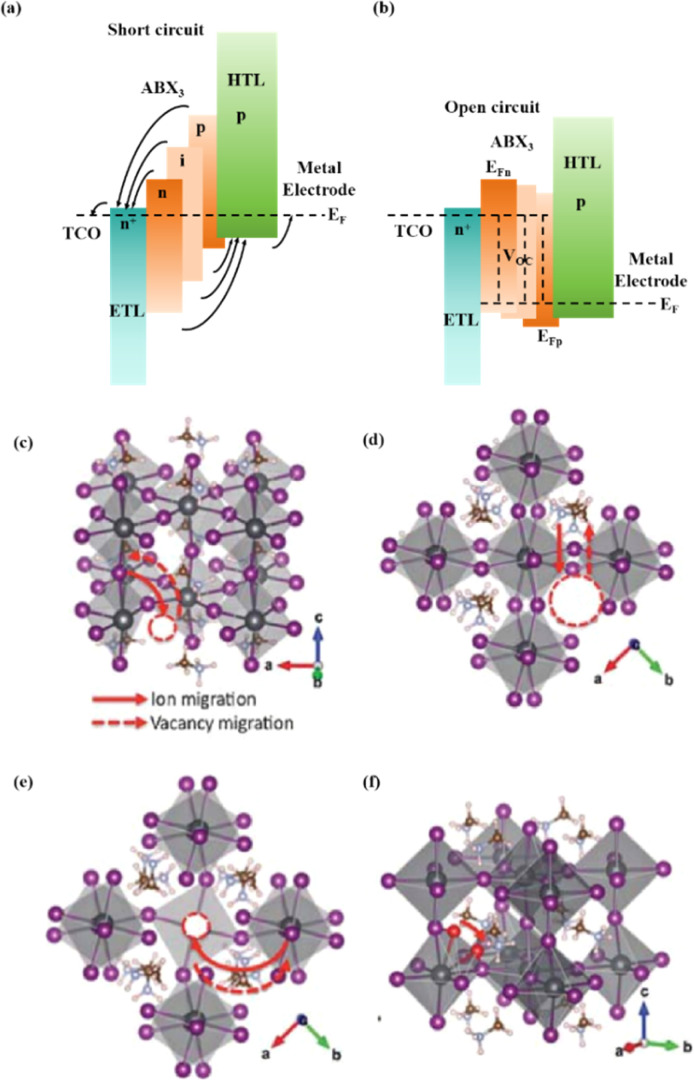
General energy band diagram in short circuit (a) and open circuit (b) for different possible perovskite conductivities, as indicated, being *E*_F_ the equilibrium Fermi lever and *E*_Fn_ and *E*_Fp_ the quasi Fermi Levels for electrons and holes, respectively. Reprinted with permission from ref. 32. Copyright 2018 John Wiley and Sons. Pathway for migration of vacancies and defects: iodine (c), methylammonium (d), lead (e) and iodine defects (f). Reprinted with permission from ref. 258. Copyright 2015 RSC Publishing.

Some studies identified or hypothesized that perovskites exhibit intrinsic p–i–n heterojunctions, whereas others have suggested p–p–n or p–n–n^+^ heterojunctions.^207^ Two possible band diagrams are shown in Fig. 29a (short circuit) and Fig. 29b (open circuit).^32^ There are no significant differences under open-circuit conditions, but under a short circuit, the photocurrent generation is quite sensitive to the properties of a few carriers. PSCs are characterized by mixed ion–electron conduction, where the types of ions that can be mobilized are associated with vacancies, ions, or gap defects within the perovskite materials.^204^ Despite the intrinsic importance of the migration and formation enthalpies in perovskite films, experimental reports on these aspects are currently lacking. A DFT study of MAPbI_3_ at room temperature showed that it contains high concentrations of iodide and MA vacancies.^253^ Some first-principles studies of MAPbI_3_ perovskites have shown that the most widely diffused anion is I^−^ (because of its higher mobility),^204,254^ suggesting that the migration of Pb and MA vacancies takes a long time. Iodide vacancy migration is the main mechanism of ion migration in perovskites. In addition, ion migration is affected by the influence of the electric field distribution on charge collection.

In drift–diffusion simulations, the drift and diffusion of anions and cations are combined with well-known drift and diffusion models of electrons and holes.^254–256^ However, incorporating these thin layers into simulations poses a computational challenge. To address this, previous studies provided models that incorporate the asymptotic expression of the Debye layer charge into a drift–diffusion simulation.^255,257^ In this model, the exponential voltage drop is associated with the Debye layers, assuming the presence of a constant compensating electric field between the layers. This model requires further discussion and includes the important feature of charge accumulation at the contact interface, which explains the large capacitance described later. Electron transport is attributed to hole and ion transport to the iodide sites and their respective vacancies.^67–69,191^ Diffusion paths along the perovskite crystals were developed to simulate vacancy and defect migration for all four explored defects (Fig. 29c–f).^258^ The vacancy (*V*) and gap defects are represented by dotted and red coils, respectively. The dotted line represents the vacancy locus and the solid line represents the migration of ions. Fig. 29c shows the formation of *V*_I_ at the equatorial position and its migration to the axial position. The *V*_I_ in the equatorial or axial positions was almost the same. However, the axial position of the *V*_Br_ was higher than the equatorial position. As shown in Fig. 29d, the inorganic scaffold was responsible for the jump in *V*_MA_ between adjacent cavities in the *ab* plane. In addition, *V*_MA_ diffused within the framework of the Pb_4_I_4_ structure. Similarly, Fig. 29e shows the in-plane migration of *V*_Pb_ along a square formed by four I and four Pb atoms.^208^ Similar to the *V*_I_, the path along the *c*-axis in Fig. 29f corresponds to MA. In the initial and/or final configuration, the interstitial I atoms are located between a pair of equatorial and/or axial I atoms of nearly the same length.^258^ The defects contribute to ion migration, implying that they undergo migration, accumulation, and propagation, resulting in irreversible degradation.^244^ Therefore, defects are crucial in controlling device stability.

**Fig. 30:**
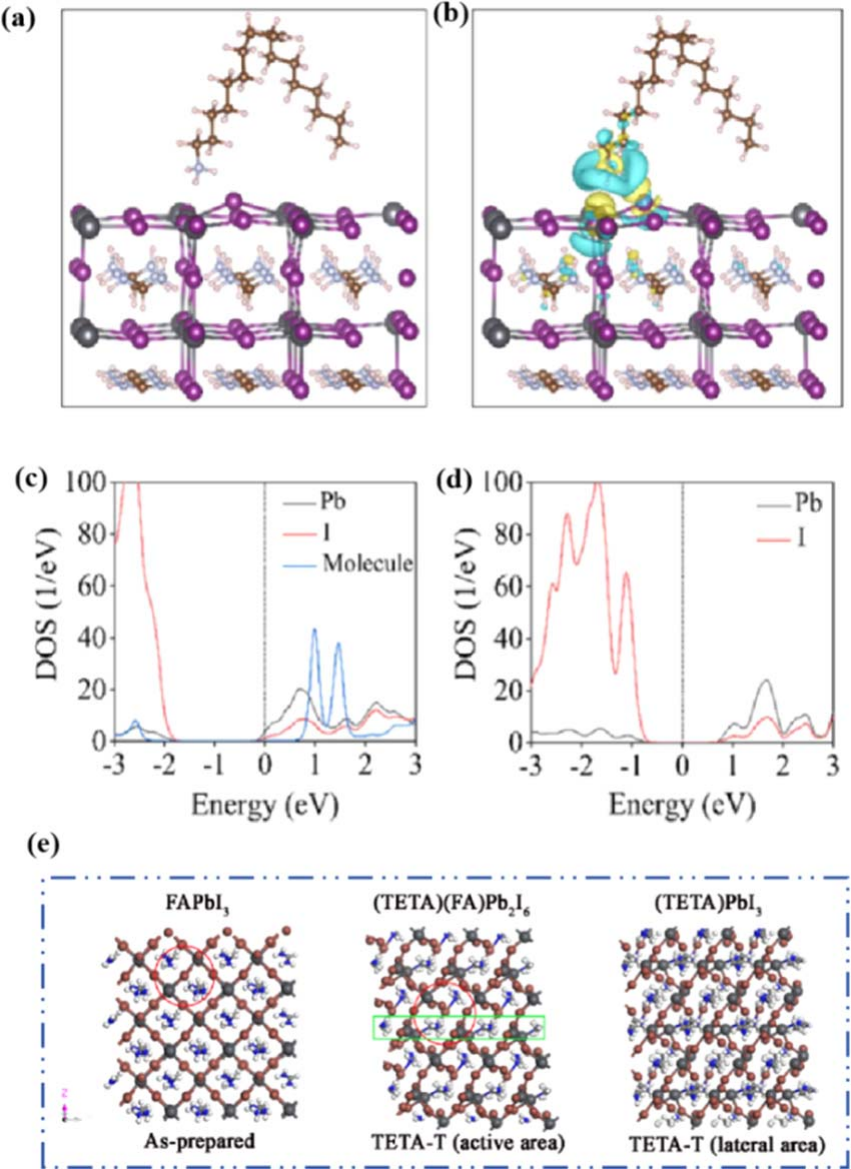
Density functional theory results: (a) relaxed structure, (b) charge density difference, and (c) partial densities of states for an oleylammonium molecule on the PbI^2−^ terminated (001) surface of FAPbI_3_. (d) Partial densities of states of the pristine PbI^2−^ terminated (001) surface of FAPbI_3_. Adapted with permission from ref. 16. Copyright 2022 The American Association for the Advancement of Science. (e) The optimal lattice structures of FAPbI_3_, (TETA)(FA)Pb_2_I_6_ and (TETA)PbI_3_ were analyzed by DFT. Adapted with permission from ref. 79. Copyright 2020 American Chemical Society.

The hidden bottom of the perovskite film and the beginning of the perovskite film crystallization are difficult to characterize because of the difficulty in tailoring.^43^ DFT is a very useful tool in the research and development of PSCs, enabling researchers to gain a deeper understanding and optimize the performance and PCE of such devices. DFT calculations are often used to simulate the crystallization parameters and electronic properties of perovskites.^18^ For example, the surface morphology of a 2D perovskite film after passivation did not change substantially.^16^ A DFT study found that the 2D perovskite passivation film exhibited stronger photoluminescence (PL) emission than that of a control 3D perovskite film with a longer attenuation life (Fig. 30a–d), owing to the inhibition of nonradiative recombination related to the surface trap state. Furthermore, DFT was used to explore the perovskite phase transition induced by treatment with TETA molecules and predict the potential products in the active and lateral regions of the TETA-treated films.^79^ As shown in Fig. 30e, the chemical bonds of Pb–I–Pb were broken after FA was replaced by TETA molecules, resulting in the separation of the [PbI_6_]^4−^ octahedron, and the lattice distortion changed from an orthogonal to a triclinic structure. These results further support the hypothesis that the inclusion of TETA molecules in the crystal structure of the perovskite leads to the transformation of the 3D structure of the perovskite into a quasi-2D material.^144^ Simulating the electronic structure of perovskite materials aids our understanding of the carrier-transport properties of the material, such as the effective masses of electrons and holes and the band structure. This knowledge is crucial for designing more efficient solar-cell materials.

**Fig. 31:**
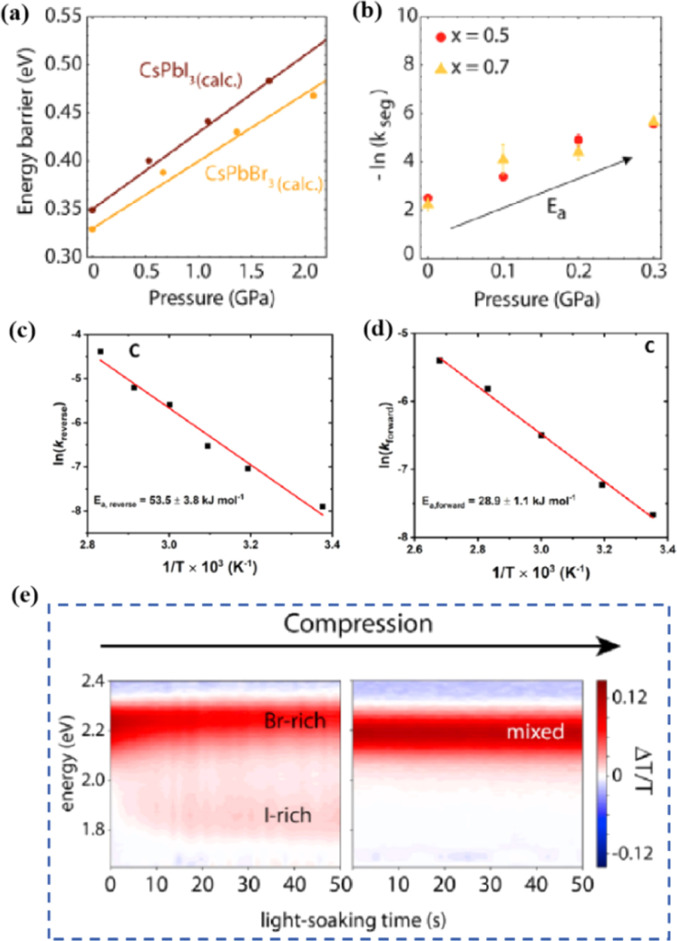
(a) Calculated activation energy for iodide and bromide diffusion in CsPbI_3_ and CsPbBr_3_ as a function of unit cell volume. (b) −ln(*k*_seg_), which is proportional to the activation energy for phase segregation, plotted against the external physical pressure applied for *x* = 0.5 and *x* = 0.7. Adapted with permission from ref. 170. Copyright 2020 American Chemical Society. Arrhenius diagram with MAPbBr_1.5_I_1.5_ mixed halide perovskite rate constant inversely proportional to temperature: (c) initial complete separation position, (d) under constant light exposure. Adapted with permission from ref. 112. Copyright 2020 American Chemical Society. (e) The relationship between halide segregation and remixing with energy, pressure, temperature, and light duration. Adapted with permission from ref. 172. Copyright 2020 Elsevier.

The DFT calculations of the model systems CsPbI_3_ and CsPbBr_3_ in Fig. 31a show the energy barrier associated with vacancy-assisted halide diffusion.^170^ The energy barriers of the two materials increased with increasing pressure; when the pressure increased from 0 to 2 GPa, the changes in the energy barriers were 0.134 eV (I) and 0.138 eV (Br), respectively. Although the changes were smaller in the pressure range of 0–0.3 GPa, their impact on migration rates could be significant owing to the exponential scaling effect. These calculations only considered the effect of pressure on the unit-cell volume in the dark. In practical hybrid materials, the strain also affects the molecular rotational dynamics, distribution of octahedral tilt, and crystal distortion. Muscarella *et al.*^170^ used the definition of the diffusion coefficient to derive the relationship between the segregation rate of ion migration (*k*_seg_) and the effective activation energy:8
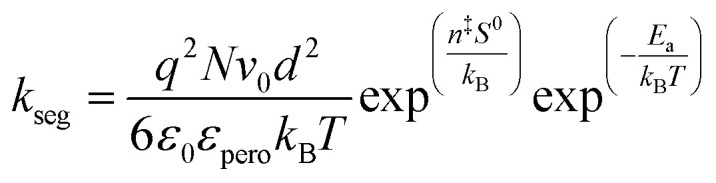
Here, Δ^‡^*S*° is the entropy change of a single ion migration step, *k*_B_ is Boltzmann's constant, *ν*_0_ is the attempted frequency of the ion transition, *d* is the distance of the ion transition, *E*_a_ is the activation energy, *q* is the electron charge, *ε*_pero_ is the perovskite dielectric constant, *ε*_0_ is the dielectric constant in vacuum, and *N* is the doping density. The natural logarithm of the migration rate, corresponding to *k*_seg_, is proportional to the activation energy of the migration process as follows:^170^

9 −ln(*k*_seg_) ∝ *E*_a_

Here, −ln(*k*_seg_) is defined as the effective activation energy 
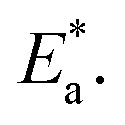
 According to this definition, the typical activation energy range for halide migration in these perovskites is approximately 100–200 MeV.^259^ As the pressure applied to all components increased to 0.3 GPa, the experimental 
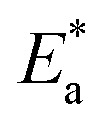
 increased by *a* factor of three as a function of the applied physical pressure (Fig. 31b). With increasing annealing temperature, the halide diffusion rate exhibited an Arrhenius relationship, giving *E*_a_ = 53/28 kJ mol^−1^ (Fig. 31c and d).^112^ A higher *E*_a_ inhibits halide segregation. Photoactivated segregation with low *E*_a_ enabled the perovskite films to overcome entropy-driven mixing and produce Br- and I-rich domains (Fig. 31e).^172^ The threshold values were qualitatively consistent with the microscopic model of phase segregation, following a linear temperature-dependent relationship, thus confirming the interaction between heat-driven mixing and light-driven ion segregation of bromide and iodide.

**Fig. 32:**
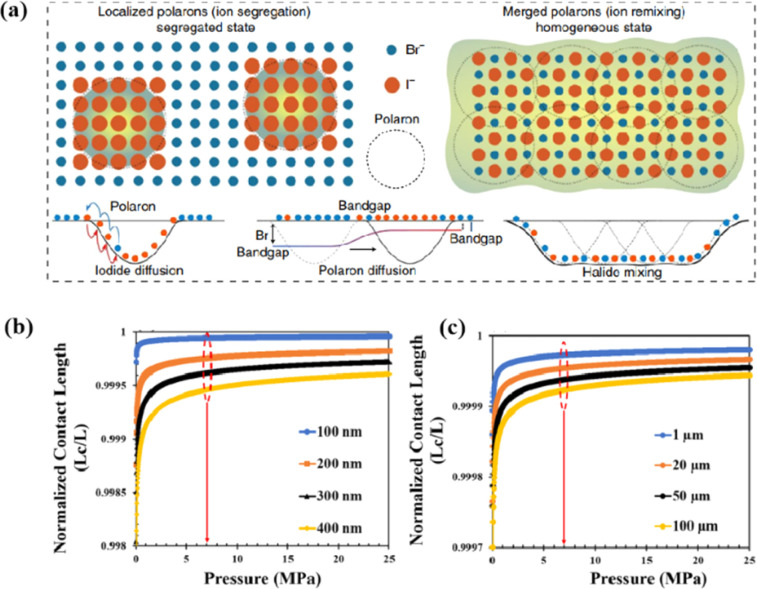
(a) Illustration of halide ion distribution for a MAPb(Br_0.8_I_0.2_)_3_ perovskite lattice in response to low carrier density (left) and high carrier density (right). Adapted with permission from ref. 209. Copyright 2020 Springer Nature. Analytical model prediction of pressure effects on contact length ratios: (b) effects of pressure on the surface contacts for different thicknesses of the films (for particle size of 1 μm) and (c) effects of pressure on surface contacts for different sizes of the particles (for a film thickness of 250 nm). Adapted with permission from ref. 31. Copyright 2020 Springer Nature.

Several mechanisms have been proposed to explain photoinduced halide segregation in mixed-halide perovskites. Initially, a temperature-dependent miscible gap was thought to be the basis of halide segregation. However, this model fails to account for the recovery of mixed phases in the absence of light or restoration of photoinduced halides at low iodine concentrations.^130^ Another model based on the anti-entropy driving force was later suggested, focusing on the segregation in the energy gap between the uniform and separated iodine-rich regions.^124^ Nevertheless, the sign of the driving force appeared to be independent of the optical excitation power and the spatial extent of the excited region. A recent study linked photoinduced halide segregation to the formation of polarons, where the deformation of the ionic perovskite lattice occurs close to the charge carrier.^131^ In this model, the change in the lattice spacing near the photoexcited carrier serves as the driving force for the separation of different halides. None of these models adequately explains the observed photoinduced halide mixing at high photon fluxes, at least in their current formulations. In contrast, Mao *et al.* developed a lattice model based on the concept of polarization-induced demixing to simulate the variation in halide distribution under broad-range illumination power.^209^ The model considers three driving forces: (1) the force that attracts I atoms locally owing to the strain gradient generated by the polaron, (2) the force that attracts the carrier/polaron to the I-rich region owing to the reduction (or funnels) in the bandgap, and (3) the force that drives the homogeneous mixing of halides in the absence of a strong strain gradient (Fig. 32a). However, many researchers have proposed that stress-induced strain compensates for photomutagenesis, thereby improving PSC performance. An analytical model was proposed to predict the surface contact between the layers associated with PSC. The deformation of the film under applied pressure is idealized by modeling the deformation of the cantilever beam around the particle,^260^ as follows:^261^10
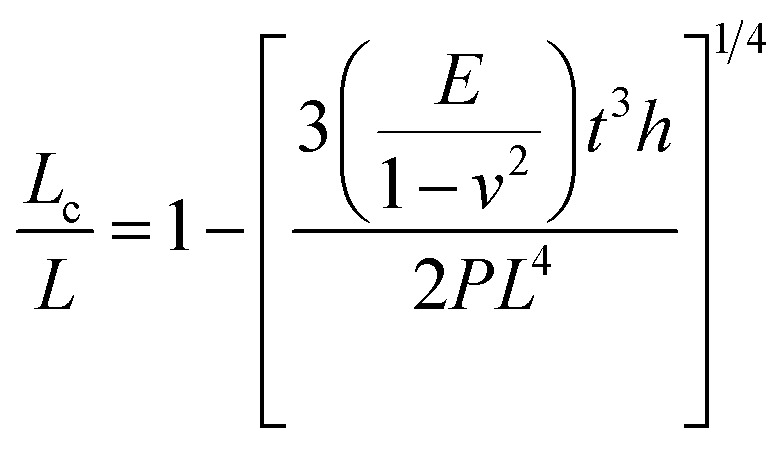
where *h* is the height of the impurity particles, *t* is the thickness of the top layer (cantilever) under compression deformation, *L*_c_ is the contact length, *L* is the cantilever beam length, *E* is Young's modulus, *v* is Poisson's ratio, and *P* is the applied pressure. The results of the surface contact analytical modeling are shown in Fig. 32b and c.^31^ For perovskite films with different thicknesses, the length ratio *L*_c_/*L* increased with increasing *P* pressure. Thinner films require less pressure for particle encapsulation, resulting in enhanced interfacial contact around the interlayer particles within thin films. Furthermore, different cleanroom conditions resulted in different particle sizes, where a reduction in particle size corresponded to an increase in the interfacial contact area. The findings of the analytical model indicate that higher pressure correlates with enhanced contact between the perovskite active layer and the adjacent layer, thereby improving charge transfer and facilitating the alignment of work functions at the interface. However, excessive pressure may lead to particle sinking^262^ potentially damaging the adjacent layers in PSCs. Excessive pressure can also cause the perovskite layer to subside into the adjacent mesoporous layer, eventually leading to short circuits. Consequently, optimal results are achieved with a judicious intermediate pressure, which enhances contact without causing detrimental effects.

## 7. Challenges and outlooks

Achieving accurate models and device simulations presents significant challenges in describing mixed ion–electron conduction. Notably, the PCEs of laminated devices remain considerably lower than those of conventional solar cells. Consequently, future efforts should concentrate on optimizing each component within the device, with a particular focus on overcoming the persistent challenge of excessively high series resistances. This requires careful selection and processing of the ETL, HTL, and additional layers introduced for lamination to minimize the resistance.

Despite the high durability of SnO_2_-based laminated structures at elevated temperatures, interfacial reactions can occur in these devices. Developing effective diffusion barriers is expected to be critical for preventing such reactions, enabling a broader selection of structures, including those incorporating more active materials, such as NiO_*x*_. In addition, inevitable buried interfaces are present in PSCs, which exhibit a high defect density, adverse strain, low crystallinity, severe ion migration, and harmful voids. Therefore, future research should focus on the fabrication of benign buried interfaces, particularly in the absence of passivation-free methods. For example, ETL materials that better match the perovskite lattice should be developed.

Vapor deposition technology, which is often overlooked because of equipment requirements, could play a crucial role in advancing PSC industrialization. Currently, PSCs produced by magnetron sputtering exhibit lower efficiencies than those produced by other methods. Exploring the application of vapor-deposition technology for industrial-scale PSC fabrication is considered a viable pathway for further development. Although studies on perovskite thin-film preparation have been conducted, achieving solvent-free, long-term, continuous, and stable deposition remains a challenge. Challenges encountered in the industrial production of devices such as organic light-emitting diodes highlight the need for improved device performance, including optimization of the crystallization process and the identification of structures compatible with solvent-free sputtering processes.

With further developments in composite engineering and the emergence of alternative materials, optimizing ion substitution at two or three sites will undoubtedly result in more stable and efficient PSCs. Given the high sensitivity of MAPbI_3_ to electron irradiation, it tends to undergo facile decomposition to form iodide. This degradation process is accompanied by subtle changes in electron diffraction, wherein certain diffraction spots are either missing or weakened, and additional spots may emerge. Consequently, atomic-resolution imaging is challenging and often leads to the misidentification of lead iodide formed after degradation as a perovskite material. Atomic-resolution imaging is a direct experimental method for visualizing structural defects affecting cell performance, including point and layer defects. However, such imaging tests are challenging with halide perovskites because of their rapid degradation under the microscopy conditions required to achieve sufficient. Therefore, improvements in micro-characterization techniques for perovskite films are imperative.

Lead-based perovskites are commonly used in PSCs because of their high PCE. Although lead-based perovskites exhibit better photocatalytic hydrogen evolution performance than lead-free perovskite materials, lead toxicity and structural instability are inevitable. These problems have not been completely overcome and are currently hindering the industrialization of PSC devices. To reduce the environmental impact of PSCs and increase their performance, future research should focus on the development of lead-free perovskite materials.

## 8. Conclusion

To facilitate the research and development of solvent-free methods for fabricating functional layers in PSCs and the integration of two half-stacks through hot-pressing techniques, this review summarized the recent advancements in the properties and strengthening mechanisms of PSCs. This review provided an overview of the preparation mechanisms involved in solvent-free methods, followed by a comprehensive summary of the PCE, stability, and interfacial morphology requirements. The main enhancement mechanisms in PSCs were systematically classified and summarized, including those based on the composition or concentration of components, pressure, temperature, doping, and passivation. Furthermore, to expedite the overall development and practical applications of PSCs, advanced characterization and simulation techniques were discussed to facilitate a comprehensive understanding of the subject. Finally, the current challenges and future developments in PSCs were discussed. This review provides new insights into improving the efficiency and stability of PSCs.

## Conflicts of interest

The authors declare that they have no known competing financial interests or personal relationships that could have appeared to influence the work reported in this paper.

## Supplementary Material
